# Nordic dietary patterns and cardiometabolic outcomes: a systematic review and meta-analysis of prospective cohort studies and randomised controlled trials

**DOI:** 10.1007/s00125-022-05760-z

**Published:** 2022-08-26

**Authors:** Paraskevi Massara, Andreea Zurbau, Andrea J. Glenn, Laura Chiavaroli, Tauseef A. Khan, Effie Viguiliouk, Sonia Blanco Mejia, Elena M. Comelli, Victoria Chen, Ursula Schwab, Ulf Risérus, Matti Uusitupa, Anne-Marie Aas, Kjeld Hermansen, Inga Thorsdottir, Dario Rahelić, Hana Kahleová, Jordi Salas-Salvadó, Cyril W. C. Kendall, John L. Sievenpiper

**Affiliations:** 1 Department of Nutritional Sciences, Temerty Faculty of Medicine, University of Toronto, Toronto, ON Canada; 2 Toronto 3D Knowledge Synthesis and Clinical Trials Unit, Toronto, ON Canada; 3 Clinical Nutrition and Risk Factor Modification Centre, St Michael’s Hospital, Toronto, ON Canada; 4 Department of Nutrition, Harvard T. H. Chan School of Public Health, Boston, MA USA; 5 Women’s College Research Institute, Women’s College Hospital, Toronto, ON Canada; 6 Joannah and Brian Lawson Centre for Child Nutrition, Temerty Faculty of Medicine, University of Toronto, Toronto, ON Canada; 7 Institute of Public Health and Clinical Nutrition, Faculty of Health Sciences, University of Eastern Finland, Kuopio, Finland; 8 Department of Medicine, Endocrinology and Clinical Nutrition, Kuopio University Hospital, Kuopio, Finland; 9 Department of Public Health and Caring Sciences, Clinical Nutrition and Metabolism, Uppsala University, Uppsala, Sweden; 10 Division of Medicine, Department of Clinical Service, Section of Nutrition and Dietetics, Oslo University Hospital, Oslo, Norway; 11 Department of Endocrinology and Internal Medicine, Aarhus University Hospital, Aarhus, Denmark; 12 Department of Clinical Medicine, Aarhus University, Aarhus, Denmark; 13 Unit for Nutrition Research, Health Science Institute, University of Iceland, Reykjavík, Iceland; 14 Landspitali – University Hospital of Iceland, Reykjavík, Iceland; 15 Vuk Vrhovac University Clinic for Diabetes, Endocrinology and Metabolic Diseases, Merkur University Hospital, Zagreb, Croatia; 16 Croatian Catholic University School of Medicine, Zagreb, Croatia; 17 Josip Juraj Strossmayer University School of Medicine, Osijek, Croatia; 18 Institute for Clinical and Experimental Medicine, Diabetes Centre, Prague, Czech Republic; 19 Physicians Committee for Responsible Medicine, Washington, DC USA; 20 Centro de Investigacion Biomedica en Red de Fisiopatología de la Obesidad y Nutrición (CIBERObn), Instituto de Salud Carlos III, Madrid, Spain; 21 Human Nutrition Department, IISPV, Universitat Rovira i Virgili, Reus, Spain; 22 College of Pharmacy and Nutrition, University of Saskatchewan, Saskatoon, SK Canada; 23 Li Ka Shing Knowledge Institute, St Michael’s Hospital, Toronto, ON Canada; 24 Division of Endocrinology and Metabolism, Department of Medicine, St Michael’s Hospital, Toronto, ON Canada; 25 Department of Medicine, Temerty Faculty of Medicine, University of Toronto, Toronto, ON Canada

**Keywords:** Cardiovascular disease, Meta-analysis, Nordic diet, Prospective cohort, Randomised controlled trial, Systematic review

## Abstract

**Aims/hypothesis:**

Nordic dietary patterns that are high in healthy traditional Nordic foods may have a role in the prevention and management of diabetes. To inform the update of the EASD clinical practice guidelines for nutrition therapy, we conducted a systematic review and meta-analysis of Nordic dietary patterns and cardiometabolic outcomes.

**Methods:**

We searched MEDLINE, EMBASE and The Cochrane Library from inception to 9 March 2021. We included prospective cohort studies and RCTs with a follow-up of ≥1 year and ≥3 weeks, respectively. Two independent reviewers extracted relevant data and assessed the risk of bias (Newcastle–Ottawa Scale and Cochrane risk of bias tool). The primary outcome was total CVD incidence in the prospective cohort studies and LDL-cholesterol in the RCTs. Secondary outcomes in the prospective cohort studies were CVD mortality, CHD incidence and mortality, stroke incidence and mortality, and type 2 diabetes incidence; in the RCTs, secondary outcomes were other established lipid targets (non-HDL-cholesterol, apolipoprotein B, HDL-cholesterol, triglycerides), markers of glycaemic control (HbA_1c_, fasting glucose, fasting insulin), adiposity (body weight, BMI, waist circumference) and inflammation (C-reactive protein), and blood pressure (systolic and diastolic blood pressure). The Grading of Recommendations, Assessment, Development and Evaluation (GRADE) approach was used to assess the certainty of the evidence.

**Results:**

We included 15 unique prospective cohort studies (*n*=1,057,176, with 41,708 cardiovascular events and 13,121 diabetes cases) of people with diabetes for the assessment of cardiovascular outcomes or people without diabetes for the assessment of diabetes incidence, and six RCTs (*n*=717) in people with one or more risk factor for diabetes. In the prospective cohort studies, higher adherence to Nordic dietary patterns was associated with ‘small important’ reductions in the primary outcome, total CVD incidence (RR for highest vs lowest adherence: 0.93 [95% CI 0.88, 0.99], *p*=0.01; substantial heterogeneity: *I*^2^=88%, *p*_Q_<0.001), and similar or greater reductions in the secondary outcomes of CVD mortality and incidence of CHD, stroke and type 2 diabetes (*p*<0.05). Inverse dose–response gradients were seen for total CVD incidence, CVD mortality and incidence of CHD, stroke and type 2 diabetes (*p<*0.05). No studies assessed CHD or stroke mortality. In the RCTs, there were small important reductions in LDL-cholesterol (mean difference [MD] −0.26 mmol/l [95% CI −0.52, −0.00], *p*_MD_=0.05; substantial heterogeneity: *I*^2^=89%, *p*_Q_<0.01), and ‘small important’ or greater reductions in the secondary outcomes of non-HDL-cholesterol, apolipoprotein B, insulin, body weight, BMI and systolic blood pressure (*p<*0.05). For the other outcomes there were ‘trivial’ reductions or no effect. The certainty of the evidence was low for total CVD incidence and LDL-cholesterol; moderate to high for CVD mortality, established lipid targets, adiposity markers, glycaemic control, blood pressure and inflammation; and low for all other outcomes, with evidence being downgraded mainly because of imprecision and inconsistency.

**Conclusions/interpretation:**

Adherence to Nordic dietary patterns is associated with generally small important reductions in the risk of major CVD outcomes and diabetes, which are supported by similar reductions in LDL-cholesterol and other intermediate cardiometabolic risk factors. The available evidence provides a generally good indication of the likely benefits of Nordic dietary patterns in people with or at risk for diabetes.

**Registration:**

ClinicalTrials.gov NCT04094194.

**Funding:**

Diabetes and Nutrition Study Group of the EASD Clinical Practice.

**Graphical abstract:**

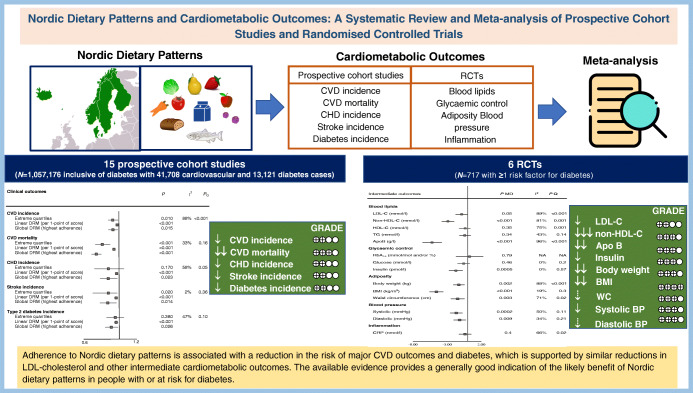

**Supplementary Information:**

The online version contains peer-reviewed but unedited supplementary material available at 10.1007/s00125-022-05760-z.

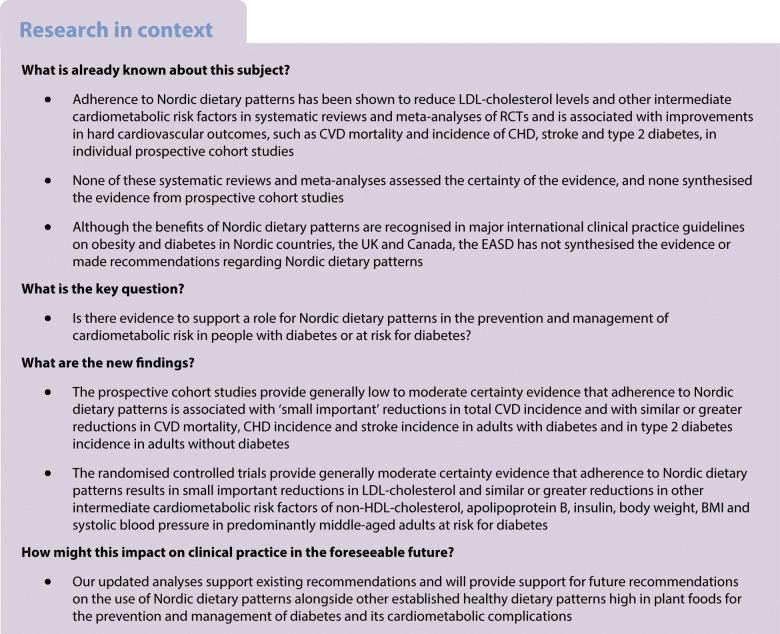



## Introduction

Several dietary patterns high in plant foods have shown advantages for managing cardiometabolic risk. Evidence from prospective cohort studies and RCTs has shown that adherence to the Mediterranean [1–9], Dietary Approaches to Stopping Hypertension (DASH) [10], Portfolio [11–19] and healthy vegetarian [20, 21] dietary patterns is associated with a lower risk of CVD and a reduction in intermediate cardiometabolic risk factors in adults with and without diabetes. The applicability of these dietary patterns to northern European countries is limited by cultural values and preferences and the availability/costs of specific foods [22–24]. Nordic dietary patterns, known variably as the Nordic diet [25], New Nordic Diet [26], healthy Nordic diet [27, 28] and Baltic Sea diet [29], include foods that are typically consumed as part of traditional Nordic diets and that are consistent with Nordic dietary guidelines [26, 30]. These foods include whole-grain cereals (especially rye, oats and barley), berries, other temperate fruits (especially apples and pears), vegetables (especially root and cruciferous vegetables), legumes, fish/shellfish, nuts and canola oil/rapeseed oil (as primary fat sources) and low-fat dairy foods [26, 27, 29, 31].

The benefits of Nordic dietary patterns have been recognised in major clinical practice guidelines on obesity [32], and diabetes [33–36]. The EASD has not reviewed the evidence or made specific recommendations on Nordic dietary patterns. Although existing systematic reviews and meta-analyses of RCTs support the benefits of Nordic dietary patterns [37–40], these syntheses did not include prospective cohort studies or assess the certainty of the evidence. To update the EASD clinical practice guidelines for nutrition therapy, the Diabetes Nutrition Study Group commissioned a systematic review and meta-analysis of prospective cohort studies and randomised trials of Nordic dietary patterns and cardiometabolic outcomes, including an assessment of the certainty of the evidence using the Grading of Recommendations, Assessment, Development and Evaluation (GRADE) approach.

## Methods

### Design

We followed the Cochrane handbook for systematic reviews of interventions [41], with results reported according to Meta-analyses of Observational Studies in Epidemiology (MOOSE) [42] and Preferred Reporting Items for Systematic Reviews and Meta-analyses (PRISMA) [43] guidelines (see electronic supplementary material [ESM] Table 1). The protocol was registered at ClinicalTrials.gov (NCT04094194).

### Data sources and searches

We searched MEDLINE, EMBASE and the Cochrane Central Register of Controlled Trials from inception to 9 March 2021 (ESM Table 2). Manual searches supplemented these database searches.

### Eligibility criteria

We included prospective cohort studies of Nordic dietary patterns and CVD and diabetes outcomes with a duration of ≥1 year, and RCTs of Nordic dietary patterns and intermediate cardiometabolic outcomes with a duration of ≥3 weeks. Populations had to have diabetes but be free of CVD for assessment of CVD outcomes, be free of diabetes for assessment of diabetes outcomes, and have diabetes or risk factors for diabetes for assessment of intermediate outcomes. Prospective cohort studies were excluded if they did not report outcome data by level of exposure using an index or scale. RCTs were excluded if they lacked a suitable comparator diet (non-isocaloric). If more than one report was available for the same study, then the report with the longest follow-up was used. There were no language restrictions (ESM Tables 3 and 4).

### Data extraction

Two reviewers (PM and EV, AJG, LC or AZ) independently extracted the data. The reviewers extracted RRs and 95% CIs for the most adjusted model from prospective cohort studies and mean differences (MDs) and SEMs from RCTs. MDs for change were preferred over end values. Missing SEMs were derived from available data using published formulae [44]. Ritz et al [45] were contacted for missing data. All disagreements were reconciled by consensus or arbitration by a senior reviewer (TAK, JLS)

### Outcomes

The primary outcome was total CVD incidence in prospective cohort studies and LDL-cholesterol in RCTs. Secondary outcomes were CVD mortality, CHD incidence and mortality, stroke incidence and mortality and type 2 diabetes incidence in prospective cohort studies, and other established lipid targets (non-HDL-cholesterol, HDL-cholesterol, triglycerides [TG], apolipoprotein B [ApoB]), markers of glycaemic control (HbA_1c_, fasting blood glucose, fasting insulin), adiposity (body weight, BMI, waist circumference [WC]) and inflammation (C-reactive protein [CRP]) and blood pressure in RCTs.

### Risk of bias

Two reviewers independently assessed risk of bias. The Newcastle–Ottawa Scale (NOS) [46] was used to assess the risk of bias in prospective cohort studies. Studies with a score of ≥6 out of 9 were considered to be of high quality. The Cochrane risk of bias tool was used to assess the risk of bias in RCTs [47] across five domains (sequence generation, allocation concealment, blinding, incomplete outcome data and selective reporting).

### Statistical analysis

Pairwise meta-analyses were conducted using Review Manager (RevMan) version 5.3 (The Nordic Cochrane Centre, The Cochrane Collaboration, Copenhagen, Denmark). All other analyses were performed using Stata 16.1 (StataCorp LP, College Station, TX, USA).

Data were pooled using DerSimonian and Laird random-effects models. RR estimates were obtained from natural log-transformed RRs and their SEMs comparing the highest with the lowest diet scores in the most adjusted models; MDs were obtained by pooling MDs and SEMs. Hazard ratios and odds ratios were treated as RRs [48]. Paired analyses were conducted for crossover trials (correlation coefficient=0.5) [49]. Fixed-effects models were used when fewer than five comparisons were available [50].

Interstudy heterogeneity was estimated using the Cochran Q test and quantified by the *I*^2^ statistic. An *I*^2^≥50% and *p*_Q_<0.1 were considered evidence of substantial heterogeneity. Sources of heterogeneity were explored in sensitivity and subgroup analyses. Sensitivity analyses were conducted by the systematic removal of each study and recalculation of the pooled estimate. Sensitivity analyses by energy control were conducted by restricting analyses to ad libitum (free intake without strict energy control) trials for adiposity outcomes. If ten or more comparisons were available, subgroup analyses were performed by meta-regression. A priori subgroup analyses were conducted by follow-up, sex, risk of bias and funding source for prospective cohort studies, and by study design, follow-up, comparator, baseline values, risk of bias, diabetes duration and funding source for RCTs.

We performed dose–response meta-analyses (DRMs) using one-stage random effects [51–53]. Nordic dietary pattern scores were scaled or standardised to the Nordic diet score, with a range between 0 and 6 [54]. Linear DRM was expressed per 1-point score, and global DRM was assessed using the non-linear association at the highest global population adherence. DRMs were planned for RCTs when adherence scores were available for six or more study comparisons.

If ten or more comparisons were available, publication bias was assessed by visual inspection of funnel plots and formal testing with the Begg and Egger tests [55, 56], with significance set at *p*<0.1. The Duval and Tweedie trim-and-fill method was used to adjust for funnel plot asymmetry [57].

### GRADE assessment

The GRADE approach [58–73] was used to assess the certainty of the evidence. The evidence from prospective cohort studies was classified initially as being of low certainty and that from RCTs was classified initially as being of high certainty, with the evidence downgraded or upgraded based on prespecified criteria. Criteria for downgrading included study limitations (high risk of bias), inconsistency (substantial unexplained interstudy heterogeneity: *I*^2^>50% and *p*_Q_<0.10), indirectness (presence of factors that limit generalisability), imprecision (95% CIs cross prespecified minimally important differences [MIDs]) and publication bias (detection of small-study effects). Criteria for upgrading included a dose–response gradient, large magnitude of effect (RR≥2 or RR≤0.5; prospective cohort studies only) and attenuation by plausible confounding. We interpreted the magnitude of the effect/association [74] based on prespecified criteria using MIDs and adapted GRADE thresholds using the following language: ‘trivial’, ‘small important’, ‘moderate’, ‘large’ and ‘very large’. At the request of the referees, we also performed a post hoc assessment of the certainty of the evidence using NutriGrade [75].

## Results

**Fig. 1:**
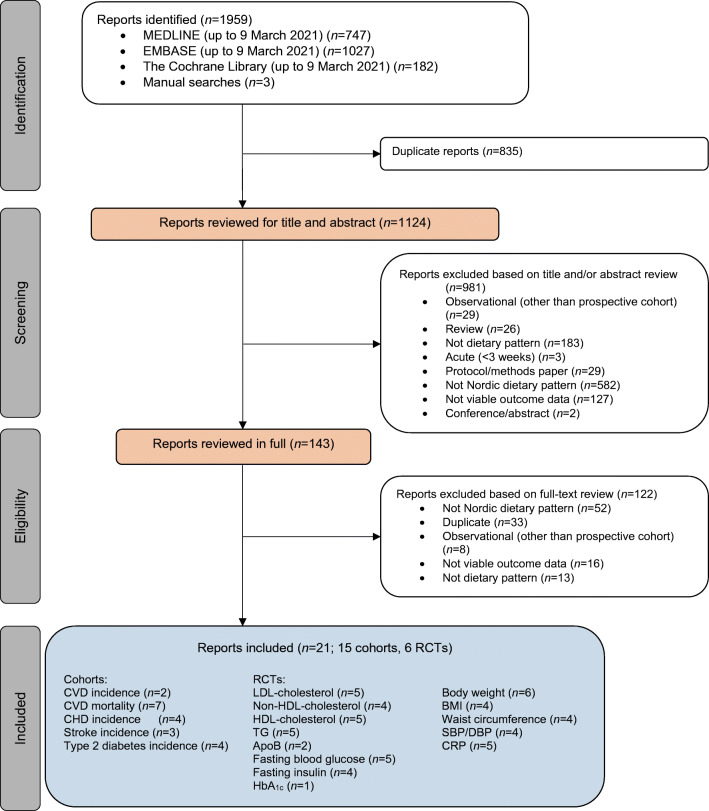
PRISMA flowchart showing the literature search

Figure 1 shows the results of the literature search. We identified 1959 reports, of which 21 met the eligibility criteria: 15 prospective cohort studies (*n*=1,057,176, with 41,708 cardiovascular events and 13,121 diabetes cases) [76–90] and six RCTs (*n*=717) [28, 91–95].

### Prospective cohort studies

#### Study characteristics

Table 1 and ESM Tables 5 and 6 show the characteristics of the prospective cohort studies included. All studies were conducted in European adult populations. The median age of participants was 49–57 years. All of the prospective cohort studies included individuals with diabetes except for the four studies (six comparisons) assessing the association of Nordic dietary patterns with type 2 diabetes incidence [82, 87–89]. The median follow-up was 13.5–17.65 years. Adherence to Nordic dietary patterns was assessed using six scores: Healthy Nordic Food Index (eight studies [76, 77, 79–81, 83, 85, 88]), diet quality index (DQI) that assesses adherence to the 2005 Swedish Nutrition Recommendations (SNR) (DQI-SNR; three studies [78, 84, 89]), Nordic diet score (one study [82]), Baltic Sea Diet Score (one study [87]), modified Baltic Sea Diet Score (one study [90]) and Danish food-based dietary guidelines (one study [86]). All studies were funded by government, university or not-for-profit sources (agency funding) except one [87], which received agency and industry funding.

**Table 1:** Summary of characteristics of the included cohorts

Outcome	Study, year	Countries, *n*	Incident cases (range)	Median (range) age, years	Median (range) follow-up, years	Dietary intake assessments (at baseline), *n*	Nordic diet exposure assessments, *n*	Method of outcome assessment, *n*	Funding^a^
CVD incidence (two cohorts, three comparisons)	Malmö Diet and Cancer cohort [78] Swedish Women’s Lifestyle and Health cohort [80]	Sweden 2	10,179 (703–8383)	49 (29–73)	17.65 (14–21.3)	7-day food records 2, FFQ 2, diet history interview 1	Healthy Nordic Food Index 1, DQI-SNR 1	Medical record linkage 3	Agency 2
CVD mortality (seven cohorts, eight comparisons)	Swedish Women’s Lifestyle and Health cohort [79] Swedish Mammography Cohort [81] Western Norway B-vitamin Intervention Trial [83] Malmö Diet and Cancer cohort [84] EPIC [85] Copenhagen General Population Study [86] Kuopio Ischaemic Heart Disease Risk Factor Study [90]	Denmark 2, France 1, Greece 1, Germany 1, Italy 1, Norway 2, Spain 1, Sweden 5, the Netherlands 1, UK 1	11,146 (171–3761)	53.75 (25–85)	14.2 (7.5–26.3)	FFQ 6, 7-day records 4, 24h recall 1	Healthy Nordic Food Index 4, DQI-SNR 2, modified Baltic Sea Diet Score 1, Danish food-based dietary guidelines 2	Medical record linkage 5, records of death 1	Agency 7
CHD incidence (four cohorts, five comparisons)	Danish Diet, Cancer and Health cohort [76] Swedish Women’s Lifestyle and Health cohort [80] EPIC-Potsdam [82] Western Norway B-vitamin Intervention Trial [83]	Denmark 1, Germany 1, Norway 1, Sweden 1	3960 (307–1669)	53.25 (29–85)	15.3 (10.8–21.3)	FFQ 4, 7-day food records 1	Nordic diet score, 1, Healthy Nordic Food Index 3	Medical record linkage 4	Agency 4
Stroke incidence (three cohorts, three comparisons)	Danish Diet, Cancer and Health cohort [77] Swedish Women’s Lifestyle and Health cohort [80] EPIC-Potsdam [82]	Denmark 1, Germany 1, Sweden 1	3302 (321–2283)	50 (29–65)	13.5 (10.8–21.3)	FFQ 3, 7-day food records 1	Nordic diet score 1, Healthy Nordic Food Index 2	Medical records linkage 2, self-report 1	Agency 3
Τype 2 diabetes incidence (four cohorts, six comparisons)	Danish Diet, Cancer and Health cohort [88] EPIC-Potsdam [82] Malmö Diet and Cancer cohort [89] Helsinki Birth Cohort Study, Health 2000 Survey [87]	Denmark 2, Sweden 2, Finland 1, Germany 1	13,121 (541–4097)	57 (25–85)	15.3 (10.8–17)	FFQ 6, 7-day records in subsample 2, diet history 2	Healthy Nordic Food Index 2, DQI-SNR 2, Baltic Sea Diet Score 1, Nordic diet score 1, Danish food-based dietary guidelines 1	Medical record linkage 5, medical record linkage and self-report 1	Agency 5, agency and industry 1

^a^Agency funding is that received from government, university or not-for-profit sources. Industry funding is that received from trade organisations that obtain revenue from the sale of products

EPIC, European Prospective Investigation into Cancer and Nutrition; FFQ, food frequency questionnaire

ESM Table 7 shows the confounding variables included in the most adjusted model for each of the cohorts included. The median (range) number of variables in the most adjusted model was 12 (7–17).

#### Risk of bias

ESM Table 8 shows the NOS scores for the cohorts included. All studies were of high quality (NOS≥6).

#### Primary outcome

**Fig. 2:**
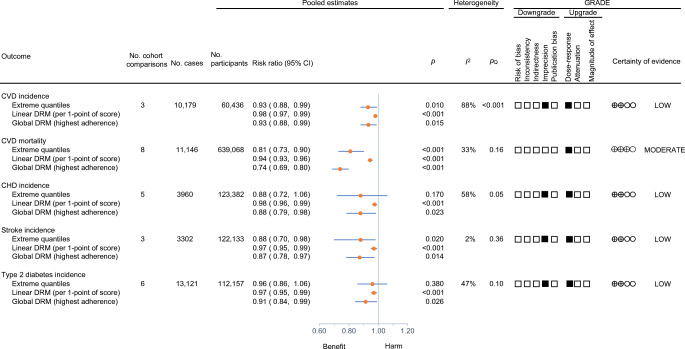
Summary plot of the association between Nordic dietary patterns and CVD, CHD, stroke and type 2 diabetes incidence and CVD mortality in prospective cohort studies. Pooled risk estimates are represented by the orange circles. The *p* values are for generic inverse variance random-effects models. Between-study heterogeneity was assessed using the Cochran Q statistic, where *p*<0.10 is considered statistically significant, and quantified by the *I*^2^ statistic, where *I*^2^≥50% is considered evidence of substantial heterogeneity [37]. Evidence from prospective cohort studies is rated as being of low certainty according to the GRADE approach and can be downgraded in five domains and upgraded in three domains. The filled black squares indicate where outcomes were downgraded and/or upgraded

**Fig. 3:**
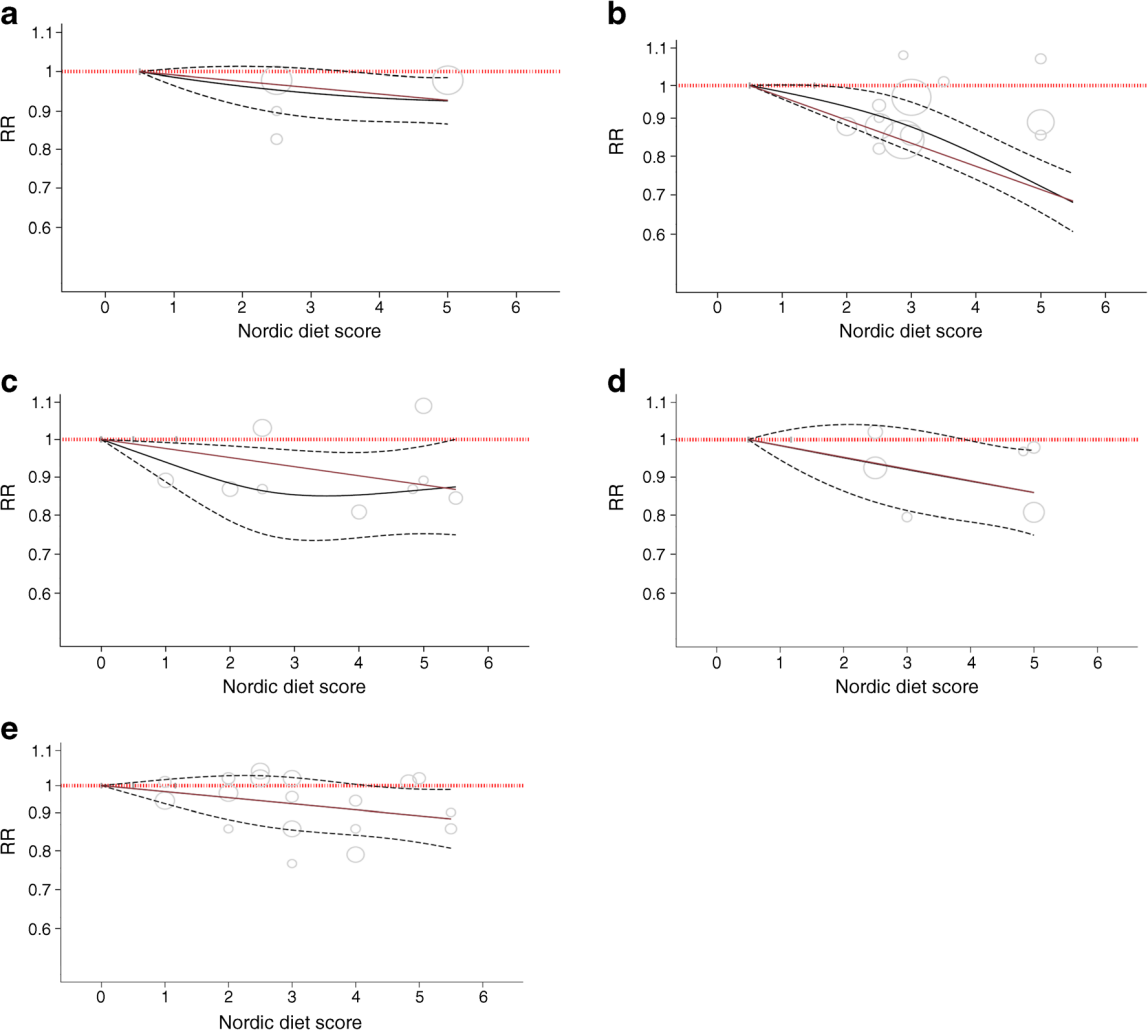
Dose–response relation between the Nordic diet score and (**a**) incidence of CVD (RR_per-diet-score_ 0.98 [95% CI 0.97, 0.99], *p*_linear_<0.001, *p*_departure-from-linearity_=0.60), (**b**) CVD mortality (RR_per-diet-score_ 0.94 [95% CI 0.93, 0.96], *p*_linear_<0.001, *p*_departure-from-linearity_=0.11), (**c**) incidence of CHD (RR_per-diet-score_ 0.98 [95% CI 0.96, 0.99], *p*_linear_<0.001, *p*_departure-from-linearity_=0.13), (**d**) incidence of stroke (RR_per-diet-score_ 0.97 [95% CI 0.95, 0.99], *p*_linear_<0.001, *p*_departure-from-linearity_=0.97) and (**e**) incidence of type 2 diabetes (RR_per-diet-score_ 0.97 [95% CI 0.95, 0.99], *p*_linear_<0.001, *p*_departure-from-linearity_=1.00). The red lines represent the linear models and the black lines represent the non-linear models. The dotted lines represent the 95% CIs for the non-linear models

Figures 2, 3a and ESM Fig. 1 show the extreme quantiles and DRMs for the association between Nordic dietary patterns and the primary outcome, total CVD incidence. Nordic dietary patterns were associated with a lower incidence of CVD (RR, 0.93 [95% CI 0.88, 0.99], *p*=0.01; substantial heterogeneity: *I*^2^=88%, *p*_Q_<0.001) comparing participants with the highest adherence with those with the lowest adherence. There was an inverse linear dose–response gradient for adherence to Nordic dietary patterns and a decrease in total CVD incidence of 2% per increase in unit of the Nordic diet score (RR 0.98 [95% CI 0.97, 0.99], *p*<0.001), with global DRM showing that adherence to Nordic dietary patterns over the global range of scores was associated with a reduction in incidence of CVD of 7% (RR 0.93 [95% CI 0.88, 0.99]).

#### Secondary outcomes

Figures 2, 3b–e and ESM Figs 2–5 show the extreme quantiles and DRMs for the association between Nordic dietary patterns and the secondary cardiometabolic outcomes. Nordic dietary patterns were associated with lower CVD mortality (RR 0.81 [95% CI 0.73, 0.90], *p*<0.001; no substantial heterogeneity: *I*^2^=33%; *p*_Q_=0.16) and stroke incidence (RR 0.88 [95% CI 0.70, 0.98], *p*=0.02; no substantial heterogeneity: *I*^2^=2%; *p*_Q_=0.36) comparing participants with the highest adherence with those with the lowest adherence. There was an inverse linear dose–response relationship for adherence to Nordic dietary patterns (*p*_departure-from-linearity_≥0.05) for all secondary outcomes, with global DRM showing that adherence to Nordic dietary patterns over the global range of scores was associated with reductions of 26% in CVD mortality (RR 0.74 [95% CI 0.69, 0.80]) and reductions of 12%, 13% and 9% in the incidence of CHD (RR 0.88 [0.79, 0.98]), stroke (RR 0.87 [0.78, 0.97]) and type 2 diabetes (RR 0.91 [0.84, 0.99]), respectively. No studies reported CHD or stroke mortality outcomes.

#### Sensitivity analyses

ESM Table 9 shows the results of the influence analyses. The systematic removal of seven individual cohort comparisons altered several findings: the significance of the summary estimate changed from non-significant to significant for CHD incidence; the evidence of substantial heterogeneity was partially or fully explained for incidence of CVD, CHD and type 2 diabetes; and the significance of the summary estimate was lost for incidence of CVD and stroke.

#### Subgroup analyses

No subgroup analyses were undertaken as fewer than ten cohort comparisons were available per outcome.

#### Publication bias

No publication bias analyses were undertaken as fewer than ten cohort comparisons were available per outcome.

### Randomised controlled trials

#### Study characteristics

Table 2 and ESM Table 10 show the characteristics of the six RCTs included [28, 91–95]. All of the trials were conducted in Europe and had a parallel design. Participants had a median age of 47.65–53.7 years and one or more risk factor for diabetes (overweight or obese [three trials], the metabolic syndrome [one trial], dyslipidaemia [one trial] or high cardiovascular risk [one trial]). Median follow-up was 12–48 weeks. Nordic dietary pattern interventions varied and comprised the New Nordic Diet (one trial [94]), new Nordic recommendations (two trials [91, 93]), Danish official dietary guidelines (one trial [92]) and healthy Nordic diet (two trials [28, 95]). The control diets also varied and comprised general healthy eating recommendation (one trial [93]), usual Western diet (one trial [28]) and usual/habitual Nordic diet (four trials [91, 92, 94, 95]). Feeding control ranged from the provision of dietary advice to the provision of some meals. Three trials received agency funding and three received agency and industry funding.

**Table 2:** Summary of characteristics of the included RCTs

Cardiometabolic risk factor	Total no. of trials	Total *n*	Median (range) sample size	Metabolic phenotypes: no. of trials	Median (range) age, years^a^	Median (range) follow-up, weeks	Trial design: no. of trials	Countries: no. of trials	Interventions: no. of trials	Comparators: no. of trials	Energy control: no. of trials	Feeding/compliance: no. of trials	Funding^b^	Overall ROB: no. of trials
LDL-cholesterol (mmol/l)	5 [28, 91, 92, 94, 95]	606	134 (73–166)	OB 3, OW 1, MetS 1, HC 1, IHD risk 4	50.5 (18–66)	24 (6–26)	Pa 5	Denmark 2, Sweden 1, Iceland 1, Finland 1	NNR 1, NND 1, HND 2, OD 1	ADD 2, UND 1, UWD 1, HD 1	Ad lib 4, isocaloric 1	Supp 2, Supp to ND 1, DA 2	A+I 3, A 2	Low 3, unclear 2
Non-HDL-cholesterol (mmol/l)	4 [28, 91, 92, 95]	374	93 (49–166)	OB 3, OW 1, MetS 1, HC 1	47.65 (18–66)	24 (6–26)	Pa 4	Denmark 3, Sweden 1	NNR 1, NND 1, HND 1, OD 1	ADD 2, UWD 1, HD 1	Ad lib 2, isocaloric 1, negative 1	Supp 2, Supp to ND 1, DA 1	A+I 2, A 2	Low 2, unclear 2
HDL-cholesterol (mmol/l)	5 [28, 91, 92, 94, 95]	606	134 (73–166)	OB 2, OW 1, MetS 1, mild HC 1; IHD risk factor 1	50.5 (18–66)	24 (6–26)	Pa 5	Denmark 4, Sweden 2, Iceland 1, Finland 1	NNR 1, NND 1, HND 2, OD 1	ADD 2, UND 1, UWD 1, HD 1	Ad lib 4, isocaloric 1	Supp 2, Supp to ND 1, DA 2	A+I 3, A 2	Low 3, unclear 2
TG (mmol/l)	5 [28, 91, 92, 94, 95]	606	134 (73–166)	OB 2, OW 1, MetS 1, mild HC 1, IHD risk factor 1	49.2 (18–66)	24 (6–26)	Pa 5	Denmark 3, Sweden 2, Iceland 1, Finland 1	NNR 1, NND 1, HND 2, OD 1	ADD 2, UND 1, UWD 1, HD 1	Ad lib 4, isocaloric 1	Supp 2, Supp to ND 1, DA 2	A+I 3, A 2	Low 3, unclear 2
ApoB (mmol/l)	2 [28, 95]	252	126 (86–166)	MetS 1, HC 1	53.7 (25–65)	12 (6–18)	Pa 2	Denmark 1, Sweden 2, Iceland 1, Finland 1	HND 2	UND 1, UWD 1	Ad lib 1, isocaloric 1	Supp 1, Supp to ND 1, DA 1	A+I 1, A 1	Low 1, unclear 1
Fasting blood glucose (mmol/l)	5 [28, 91, 92, 94, 95]	606	134 (73–166)	OB 2, OW 1, MetS 1, HC 1, IHD risk 1	49.2 (18–66)	24 (6–26)	Pa 5	Denmark 5, Sweden 2, Iceland 1, Finland 1	NNR 1, NND 1, HND 2, OD 1	ADD 2, UND 1, UWD 1, HD 1	Ad lib 4, isocaloric 1	Supp 3, Supp to ND 1, DA 3	A+I 3, A 2	Low 3, unclear 2
Fasting insulin (pmol/l)	4 [28, 91, 92, 94]	440	110 (73–147)	OB 2, OW 1, HC 1, IHD risk 1	49.2 (27.3–51.8)	24 (6–26)	Pa 4	Denmark 3, Sweden 1	NNR 1, NND 1, HND 1, OD 1	ADD 2, UWD 1, HD 1	Ad lib 4	Supp 2, Supp to ND 1, DA 1	A+I 2, A 2	Low 3, unclear 1
HbA_1c_ (mmol/mol and/or %)	1 [92]	145	145	IHD risk 1	50.5 (30–65)	48	Pa 1	Denmark 1	OD 1	HD 1	Ad lib 1	DA 1	A 1	Low 1
Body weight (kg)	6 [28, 91–95]	706	117 (86–166)	OB 2, OW 2, MetS 1, mild HC 1, IHD risk factor 1	49.2 (18–66)	24 (6–48)	Pa 6	Denmark 4, Sweden 3, Iceland 1, Finland 1	NNR 2, NND 1, HND 2, OD 1	ADD 2, UND 1, UWD 1, GHE 1, HD 1	Ad lib 4, isocaloric 1, negative 1	Supp 2, Supp to ND 1, DA 4	A+I 3, A 3	Low 4, unclear 2
BMI (kg/m^2^)	4 [28, 91–93]	393	93 (49–100)	OB 1, OW 2, mild HC 1, IHD risk factor 1	40.9 (18–65)	18 (6–24)	Pa 4	Denmark 2, Sweden 2	NNR 2, HND 1, OD 1	ADD 1, UWD 1, GHE 1, HD 1	Ad lib 3, negative 1	Supp 1, Supp to ND 1, DA 2	A+I 1, A 3	Low 3, unclear 1
Waist circumference (cm)	4 [28, 91–93]	454	96.8 (92.3–105.3)	OB 2, OW 2, IHD risk factor 1	40.9 (18–66)	24 (12–26)	Pa 4	Denmark 3, Sweden 1	NNR 2, NND 1, OD 1	ADD 2, GHE 1, HD 1	Ad lib 3, negative 1	Supp 1, Supp to ND 1, DA 3	A+ I 1, A 3	Low 3, unclear 1
SBP (mmHg)	4 [28, 92, 94, 95]	533	140.5 (86–166)	OB 1, MetS 1, mild HC 1, IHD risk factor 1	52.2 (25–65)	21 (6–26)	Pa 4	Denmark 2, Sweden 2, Iceland 1, Finland 1	NND 1, HND 2, OD 1	ADD 1, UND 1, UWD 1, HD 1	Ad lib 3, isocaloric 1	Supp 1, Supp to ND 2, DA 1	A+I 2, A 2	Low 3, unclear 1
DBP (mmHg)	4 [28, 92, 94, 95]	533	140.5 (86–166)	OB 1, MetS 1, mild HC 1, IHD risk factor 1	52.2 (25–65)	21 (6–26)	Pa 4	Denmark 2, Sweden 2, Iceland 1, Finland 1	NND 1, HND 2, OD 1	ADD 1, UND 1, UWD 1, HD 1	Ad lib 3, isocaloric 1	Supp 1, Supp to ND 2, DA 1	A+I 2, A 2	Low 3, unclear 1
CRP (nmol/l)^c^	5 [28, 91, 92, 94, 95]	606	134 (86–166)	OB 2, OW 1, MetS 1, mild HC 1, IHD risk factor 1	50.5 (6–24)	24 (6–26)	Pa 5	Denmark 4, Sweden 2, Iceland 1, Finland 1	NNR 1, NND 1, HND 2, OD 1	ADD 2, UND 1, UWD 1, HD 1	Ad lib 4, isocaloric 1	Supp 1, Supp to ND 2, DA 5	A+I 3, A 2	Low 3, unclear 2

^a^The range represents the range of the mean age in the trials

^b^Agency funding is that received from government, university or not-for-profit sources. Industry funding is that received from trade organisations that obtain revenue from the sale of products

^c^Five out of six studies reported high-sensitivity CRP levels

A, agency; ADD, average Danish diet; Ad lib, ad libitum; DA, dietary advice; DODG, Danish official dietary guidelines; GHE, general healthy eating; HC, hypercholesterolaemia; HD, habitual diet; HND, healthy Nordic diet; I, industry; IHD, ischaemic heart disease; MetS, the metabolic syndrome; ND, Nordic diet; NND, New Nordic Diet; NNR, Nordic nutrition recommendations; OB, obese; OW, overweight; Pa, parallel design; ROB, risk of bias; Supp, supplemented; UND, usual Nordic diet; UWD, usual Western diet

#### Risk of bias

ESM Fig. 6 shows the summary and individual Cochrane risk of bias assessments for the included RCTs. Most RCTs were judged as having a low or unclear risk of bias across the five domains. Although three trials were rated as having a high risk of bias in one of the five domains, overall there was no evidence of serious risk of bias.

#### Primary outcome

**Fig. 4:**
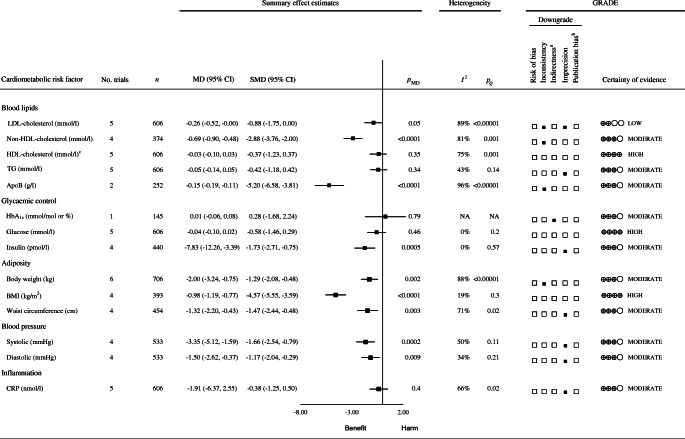
Summary plot of the effect of Nordic dietary patterns on cardiometabolic risk factors in RCTs. Data are expressed as weighted MDs with 95% CIs using the generic inverse variance method modelled by random effects ( five trials available) or fixed effects (fewer than five trials available). To allow the pooled effect estimates for each endpoint to be displayed on the same axis, MDs were transformed to standardised mean differences (SMDs). The pseudo 95% CI for each transformed SMD was derived directly from the original MD and 95% CI. Between-study heterogeneity was assessed by the Cochran Q statistic, where *p*<0.10 is considered statistically significant, and quantified by the *I*^2^ statistic, where *I*^2^≥50% is considered evidence of substantial heterogeneity [61]. Evidence from RCTs is rated as being of high certainty according to the GRADE approach and can be downgraded in five domains. The filled black squares indicate where outcomes were downgraded. ^a^Although all studies were conducted in Nordic countries and in those who were overweight or obese, we did not downgrade the evidence in this domain as there is no biological reason why the results would differ in other populations. ^b^Unable to assess publication bias because of fewer than ten studies per outcome. ^c^Because of the difference in directionality of HDL-cholesterol compared with the other outcomes with regard to the signal for benefit or harm, the signs for the MD and SMD were changed. To convert total cholesterol, LDL-cholesterol and HDL-cholesterol to mg/dl, multiply by 38.67; to convert TG to mg/dl, multiply by 88.57; to convert blood glucose to mg/dl, multiply by 18.02; to convert CRP to mg/l, multiply by 0.105. NA, not available

Figure 4 and ESM Fig. 7 show the effects of Nordic dietary patterns on the intermediate primary outcome LDL-cholesterol. Adherence to Nordic dietary patterns was associated with a reduction in LDL-cholesterol compared with control diets (MD −0.26 mmol/l [95% CI −0.52, −0.00], *p*_MD_=0.05; substantial heterogeneity: *I*^2^=89%, *p*_Q_<0.001). Linear or non-linear dose–responses could not be assessed.

#### Secondary outcomes

Figure 4 and ESM Figs 8–20 show the effects of Nordic dietary patterns on the intermediate secondary outcomes. Compared with control diets, Nordic dietary patterns were associated with reductions in non-HDL-cholesterol (−0.69 mmol/l [95% CI −0.9, −0.48], *p*_MD_ <0.01; substantial heterogeneity: *I*^2^=81%, *p*_Q_<0.01), ApoB (−0.15 g/l [95% CI −0.19, −0.11], *p*_MD_<0.01; substantial heterogeneity: *I*^2^=96%, *p*_Q_<0.01), body weight (−2.00 kg [95% CI −3.24, −0.75], *p*_MD_=0.002; substantial heterogeneity: *I*^2^=88%, *p*_Q_<0.01), BMI (−0.98 kg/m^2^ [95% CI −1.19, −0.77], *p*_MD_<0.01; no substantial heterogeneity: *I*^2^=19%, *p*_Q_=0.3); WC (−1.32 cm [95% CI −2.20, −0.43], *p*_MD_=0.003; substantial heterogeneity: *I*^2^=71%, *p*_Q_=0.02); insulin (−7.83 pmol/l [95% CI −12.26, −3.39], *p*_MD_<0.01; no substantial heterogeneity: *I*^2^=0%, *p*_Q_=0.57), systolic blood pressure (SBP; −3.35 mmHg [−5.12, −1.59], *p*_MD_<0.01; substantial heterogeneity: *I*^2^=50%, *p*_Q_=0.11) and diastolic blood pressure (DBP; −1.50 mmHg [−2.62, −0.37], *p*_MD_=0.009; no substantial heterogeneity: *I*^2^=34%, *p*_Q_=0.21). There were no effects on the other intermediate cardiometabolic outcomes. Linear or non-linear dose–responses could not be assessed.

#### Sensitivity analyses

ESM Table 11 shows selected sensitivity analyses in which systematic removal of individual trials altered the results. Systematic removal of individual trials resulted in the following: loss of significance for LDL-cholesterol, ApoB and WC, although the pooled effect estimates still favoured Nordic diets; change in the pooled effect estimate from non-significant to a significant decrease for TG; and explanation of the substantial heterogeneity for LDL-cholesterol, non-HDL-cholesterol, TG, HDL-cholesterol, BMI, WC, DBP and CRP.

ESM Fig. 21 shows the results of the sensitivity analysis including only ad libitum trials. Removal of two trials (those by Uusitupa et al [95] [isocaloric trial] and Huseinovic et al [93] [negative energy balance trial]) resulted in reductions in body weight (MD −2.16 kg [95% CI −3.51 to −0.82 mmol/l], *p*_MD_=0.002; substantial heterogeneity: *I*^2^=82%, *p*_Q_=0.001), BMI (MD −0.85 kg/m^2^ [95% CI −1.31 to −0.40 mmol/l], *p*_MD_<0.01; no substantial heterogeneity: *I*^2^=37%, *p*_Q_=0.20) and WC (−1.32 cm [95% CI −3.49, 0.84], *p*_MD_=0.23; substantial heterogeneity: *I*^2^=78%, *p*_Q_=0.01) without changing the significance or the magnitude of the effect, except for BMI, for which the magnitude of the reduction was decreased.

#### Subgroup analyses

No subgroup analyses were undertaken as fewer than ten trial comparisons were available for any outcome.

#### Publication bias

No publication bias analyses were undertaken as fewer than ten trial comparisons were available for any outcome.

#### Medication use

Of the six trials, only two reported medication use [94, 95]. In one trial, one participant in the New Nordic Diet group and one in the average Danish diet group began antihypertensive medication during the study [94], while in the other trial no changes in dosage of antihypertensive and lipid-lowering medication were allowed during the study [95].

#### Adverse events

Two trials assessed adverse events [28, 92]. No adverse events were reported.

#### Acceptability

No trials assessed the acceptability of Nordic dietary patterns. Four trials assessed adherence to the Nordic dietary pattern, which was reported as satisfactory or high [28, 91, 94, 95].

### GRADE assessments

ESM Table 12 summarises the GRADE assessments of the associations between Nordic dietary patterns and CVD outcomes in prospective cohorts. The certainty of evidence for the primary outcome, total CVD incidence (small important reduction), was graded as low owing to downgrades for imprecision and inconsistency and an upgrade for dose–response gradient. For the secondary outcomes, the certainty of evidence was graded as moderate for CVD mortality (moderate reduction) owing to an upgrade for dose–response gradient and no downgrades, and low for CHD (small important reduction), stroke (small important reduction) and type 2 diabetes (small important reduction) owing to downgrades for imprecision and upgrades for dose–response gradient in all cases. NutriGrade assessments gave the same ratings as the GRADE approach for four out of five (80%) outcomes, and a lower rating for the remaining outcome (20%) (ESM Table 13).

ESM Table 14 shows the GRADE assessments conducted for the effect of Nordic dietary patterns on cardiometabolic risk factors in RCTs. The certainty of evidence for the primary outcome, LDL-cholesterol (small important reduction), was graded as low owing to downgrades for inconsistency and imprecision. The certainty of evidence for the secondary outcomes was graded as high for HDL-cholesterol (no effect), BMI (moderate reduction) and blood glucose (no effect) in the absence of downgrades, moderate for non-HDL-cholesterol (large reduction), ApoB (moderate reduction) and body weight (moderate reduction) owing to downgrades for inconsistency, and moderate for TG (no effect), insulin (small important reduction), WC (trivial reduction), SBP (small important reduction), DBP (trivial reduction) and CRP (no effect) owing to downgrades for imprecision. Compared with the GRADE approach, NutriGrade assessments gave the same ratings for one of the 14 (7%) outcomes and lower ratings for 13 of 14 (93%) outcomes (ESM Table 15).

## Discussion

We conducted a comprehensive systematic review and meta-analysis of Nordic dietary patterns and cardiometabolic outcomes, including 15 prospective cohort studies (*n*=1,057,176 with 41,708 cardiovascular events and 13,121 diabetes cases) and six RCTs (*n*=717). We observed that Nordic dietary patterns were associated with a small important reduction in the primary clinical outcome of CVD incidence (7% by global DRM), with similar or greater reductions in the secondary clinical outcomes of CVD mortality (26% by global DRM), CHD incidence (12% by global DRM) and stroke incidence (13% by global DRM) in adults with diabetes, and type 2 diabetes incidence (9% by global DRM) in adults without diabetes. These reductions were supported by reductions in intermediate cardiometabolic outcomes in adults with one or more risk factor for diabetes. Nordic dietary patterns resulted in small important reductions in the primary intermediate outcome LDL-cholesterol (−0.26 mmol/l) and similar or greater reductions in the secondary intermediate outcomes of non-HDL-cholesterol (−0.69 mmol/l), ApoB (−0.15 g/l), body weight (−2 kg), insulin (−7.83 pmol/l) and SBP (−3.35 mmHg). Other secondary outcomes showed trivial reductions or no effect.

### Findings in the context of the literature

We are not aware of any previous systematic reviews and meta-analyses of prospective cohort studies of Nordic dietary patterns; however, our findings agree with those of previous systematic reviews and meta-analyses of key foods that are emphasised as being part of Nordic dietary patterns. Systematic reviews and meta-analyses of prospective cohort studies have demonstrated that total fruit and vegetable intake is associated with reductions in CVD, CHD and stroke incidence and/or mortality, with the greatest benefits found for certain root vegetables (i.e. carrots) and cruciferous vegetables (including cabbage) [96]. Other key components of Nordic dietary patterns, including whole grains (oats and rye) [97, 98], fish [99] and legumes [100], have been associated with reductions in CVD, stroke and cardiovascular mortality and/or incidence of type 2 diabetes.

Our findings also agree with and expand on those of several previous systematic reviews and meta-analyses of RCTs of Nordic dietary patterns and intermediate cardiometabolic outcomes. Three systematic reviews and meta-analyses of RCTs found reductions in SBP associated with Nordic dietary patterns [37, 101, 102]. One systematic review and meta-analysis of RCTs showed similar reductions in blood insulin levels but not blood glucose levels [38]. A second showed similar reductions in body weight but not BMI and WC [39, 103]. A third showed no effect of Nordic dietary patterns on inflammation, which agrees with our finding of no effect on CRP [40], although a single RCT did report interleukin-1 receptor antagonism [95].

There are several possible explanations for the observed benefits of Nordic dietary patterns. One is the concordance of Nordic dietary patterns with Mediterranean, DASH, Portfolio, healthy vegetarian and low glycaemic index/load dietary patterns, which are associated with improvements in clinical cardiometabolic outcomes and intermediate cardiometabolic outcomes [3, 10–12, 104–107]. Key foods shared with these other dietary patterns [27, 30] have also been shown to improve intermediate cardiometabolic outcomes. These foods include viscous fibres [108, 109] from oats and barley [110, 111], temperate fruit and berries [112, 113], nuts [103, 114–116] and legumes [117–125]. Another possible explanation for the observed benefits of Nordic dietary patterns is because of weight loss induced by the interventions. Most of the included RCTs, however, adjusted for weight [28, 94] or BMI [91, 93], indicating that effects were largely independent of weight loss.

### Strengths and limitations

The present systematic review and meta-analysis has several strengths. It provides a comprehensive synthesis of the currently available evidence on the potential role of Nordic dietary patterns in both patient-important and surrogate CVD outcomes. We used a systematic search strategy to capture all pertinent prospective cohort studies and RCTs. We explored dose–responses in prospective cohorts, which highlighted a significant linear protective association of Nordic dietary patterns with CVD outcomes. We assessed the certainty of the evidence using the GRADE approach and performed a post hoc analysis using NutriGrade (although the latter was not used to inform our assessment of the certainty of the evidence as it was not prespecified or endorsed by the guidelines committee).

Several limitations were identified in the available evidence. First, there was evidence of serious inconsistencies. We observed substantial unexplained heterogeneity in the primary outcomes, total CVD incidence in the prospective cohort studies and LDL-cholesterol in the RCTs, and in several of the secondary outcomes in the RCTs. Second, there was evidence of serious imprecision. We observed imprecision in the primary outcomes, total CVD incidence in the prospective cohort studies and LDL-cholesterol in the RCTs, and in several of the secondary outcomes. Finally, there was some evidence of indirectness. Different criteria were used to define Nordic dietary patterns, which may have contributed to the heterogeneity observed. We downgraded the evidence for HbA_1c_ because of serious indirectness, as data were available from only a single RCT of a single Nordic dietary pattern (Danish official dietary guidelines), limiting generalisability to other Nordic dietary patterns. We did not, however, downgrade the evidence for other outcomes because of the use of different definitions of Nordic dietary patterns, as the evidence appeared robust to the different definitions. Another potential source of indirectness was the inability to isolate the effects/associations in diabetes. Prospective cohort studies did not provide subgroup data by diabetes status, and none of the RCTs included individuals with diabetes. We did not downgrade for indirectness here, as the prospective cohort studies did include a representative proportion of individuals with diabetes and the RCTs included individuals at risk for diabetes. The key components of Nordic dietary patterns have also been shown individually to lower cardiometabolic risk factors reliably in people with diabetes [108–126].

### Implications

Dietary interventions remain the cornerstone of type 2 diabetes and CVD prevention and management [127–129]. Clinical practice guidelines for obesity, type 2 diabetes and CVD have shifted from focusing on single nutrients to focusing on dietary patterns [127–129]. Nordic dietary patterns ( ≥25% energy as whole grains, ≥175g/day of temperate fruits, ≥150–200g/day of berries, ≥175g/day of vegetables, legumes and canola oil, three or more servings/week of fatty fish, two or more servings/day of low-fat dairy products) [95] have important similarities (with some differences [130, 131]) to other established dietary patterns that are high in plant foods such as the Mediterranean, DASH, Portfolio and vegetarian dietary patterns. Population intakes of Nordic countries, as well as other European countries, Canada and the USA, do not meet the targets for these other dietary patterns [125, 132–135]. Nordic dietary patterns may provide a promising alternative to help individuals in Nordic countries and elsewhere achieve the cardiometabolic benefits of dietary interventions. This approach may have impacts beyond health [136, 137]. As pointed out by the WHO Regional Office for Europe, the Nordic nutrition recommendations will be the first nutrition recommendations integrating environmental health with personal health [137].

### Conclusions

Adherence to Nordic dietary patterns is associated with generally small important reductions in major CVD outcomes and incidence of diabetes and similar or greater reductions in LDL-cholesterol and other intermediate cardiometabolic outcomes. Our confidence in the evidence is generally low to moderate, with the evidence for reductions in clinical outcomes from prospective cohort studies supported by reductions in intermediate cardiometabolic outcomes from RCTs. Although there is a need for more long-term RCTs using standard definitions of Nordic dietary patterns that assess the effects on clinical outcomes and intermediate cardiometabolic outcomes (especially HbA_1c_) in diabetes, the available evidence provides a generally good indication of the likely benefit of Nordic dietary patterns in adults with or at risk for diabetes.

## Supplementary information

ESM (PDF 991 kb)

## Data Availability

All data generated or analysed during this study are included in this published article (and its ESM information files).

## Abbreviations

ApoB: Apolipoprotein B
CRP: C-reactive protein
DASH: Dietary Approaches to Stopping Hypertension
DBP: Diastolic blood pressure
DQI-SNR: Diet quality index (DQI) that assesses adherence to the 2005 Swedish Nutrition Recommendations (SNR)
DRM: Dose–response meta-analysis
GRADE: Grading of Recommendations, Assessment, Development and Evaluation
MD: Mean difference
MID: Minimally important difference
NOS: Newcastle–Ottawa Scale
PRISMA: Preferred Reporting Items for Systematic Reviews and Meta-analyses
SBP: Systolic blood pressure
TG: Triglycerides
WC: Waist circumference
