# Supplementary material for: Nordic dietary patterns and cardiometabolic outcomes: a systematic review and meta-analysis of prospective cohort studies and randomised controlled trials
Source: Diabetologia. 2022 Aug 26;65(12):2011–31. doi: 10.1007/s00125-022-05760-z (PMC9630197; doi:10.1007/s00125-022-05760-z)
Supplement: Supplementary file 1 — (PDF 991 kb) [file 125_2022_5760_MOESM1_ESM.pdf]

SUPPLEMENTARY TABLES AND FIGURES

SUPPLEMENTARY TABLES 3

ESM Table 1. PRISMA checklist. 3

ESM Table 2. Search strategy. 6

ESM Table 3 Eligibility criteria for prospective cohort studies 13

ESM Table 4 PICOTS[a] framework for inclusion of randomized controlled trials 13

ESM Table 5. Characteristics of included cohorts. 13

ESM Table 6. Dietary scores used to assess adherence to the Nordic dietary pattern. 16

ESM Table 7. Confounding variables of included cohorts. 18

ESM Table 7. Confounding variables of included cohorts (continued). 19

ESM Table 8. Newcastle-Ottawa Scale (NOS) scores of included cohorts. 20

ESM Table 9. Selected sensitivity analyses in which the systematic removal of a cohort study altered the significance of the effect estimate or the evidence for heterogeneity. 21

ESM Table 10. Characteristics of included RCTs. 22

ESM Table 11. Selected sensitivity analyses in which the systematic removal of an individual trial altered the significance of the effect estimate or the evidence for heterogeneity. 24

ESM Table 12. GRADE assessment for the association between Nordic dietary patterns and cardiometabolic disease outcomes for prospective cohort studies. 25

ESM Table 13 NutriGRADE Assessment for association of Nordic dietary patterns with cardiometabolic outcomes in Cohort studies. 26

ESM Table 14. GRADE assessment for the effect of Nordic dietary patterns and cardiometabolic risk factors in RCTs. 28

ESM Table 15 NutriGRADE assessment for the effect of Nordic dietary patterns and cardiometabolic risk factors in RCTs30

SUPPLEMENTARY FIGURES 32

ESM Fig. 1. Forest plot of the association between the Nordic dietary patterns and CVD incidence. 32

ESM Fig. 2. Forest plot of the association between the Nordic dietary patterns and CVD mortality. 33

ESM Fig. 3. Forest plot of the association between the Nordic dietary patterns and CHD incidence. 33

ESM Fig. 4. Forest plot of the association between the Nordic dietary patterns and stroke incidence. 34

ESM Fig. 5. Forest plot of the association between the Nordic dietary patterns and type 2 diabetes mellitus incidence. .34

ESM Fig. 6. Risk of bias of included RCTs. 35

ESM Fig. 7. Forest plot of randomized controlled trials assessing the effect of Nordic diets on LDL-C. 36

ESM Fig. 8. Forest plot of randomized controlled trials assessing the effect of Nordic dietary patterns on BMI...............36

ESM Fig. 9. Forest plot of randomized controlled trials assessing the effect of Nordic dietary patterns on body weight. .37

ESM Fig. 10. Forest plot of randomized controlled trials assessing the effect of Nordic dietary patterns on waist circumference. 37

ESM Fig. 11. Forest plot of randomized controlled trials assessing the effect of Nordic dietary patterns on HDL-C. 38

ESM Fig. 12. Forest plot of randomized controlled trials assessing the effect of Nordic dietary patterns on Non-HDL-C. .38

ESM Fig. 13. Forest plot of randomized controlled trials assessing the effect of Nordic dietary patterns on triglycerides.39

ESM Fig. 14. Forest plot of randomized controlled trials assessing the effect of Nordic dietary patterns on ApoB. 39

ESM Fig. 15. Forest plot of randomized controlled trials assessing the effect of Nordic dietary patterns on systolic blood pressure. 40

ESM Fig. 16. Forest plot of randomized controlled trials assessing the effect of Nordic dietary patterns on diastolic blood pressure. 40

ESM Fig. 17. Forest plot of randomized controlled trials assessing the effect of Nordic dietary patterns on fasting blood glucose. 41

ESM Fig. 18. Forest plot of randomized controlled trials assessing the effect of Nordic dietary patterns on fasting blood insulin. 41

ESM Fig. 19. Forest plot of randomized controlled trials assessing the effect of Nordic dietary patterns on HbA1c..........42

ESM Fig. 20. Forest plot of randomized controlled trials assessing the effect of Nordic dietary patterns on inflammation.42

ESM Fig. 21. Influence analysis plots of ad libitum randomized controlled trials assessing the effect of Nordic dietary patterns on adiposity markers. 43

SUPPLEMENTARY TABLES

ESM Table 1. PRISMA checklist.

Section and Topic	Item #	Checklist item	Location where item is reported
**TITLE**			
Title	1	Identify the report as a systematic review.	Page 1
**ABSTRACT**			
Abstract	2	See the PRISMA 2020 for Abstracts checklist.	Page 1
**INTRODUCTION**			
Rationale	3	Describe the rationale for the review in the context of existing knowledge.	Page 3
Objectives	4	Provide an explicit statement of the objective(s) or question(s) the review addresses.	Page 3
**METHODS**			
Eligibility criteria	5	Specify the inclusion and exclusion criteria for the review and how studies were grouped for the syntheses.	Page 3, Figure 1
Information sources	6	Specify all databases, registers, websites, organisations, reference lists and other sources searched or consulted to identify studies. Specify the date when each source was last searched or consulted.	Page 3
Search strategy	7	Present the full search strategies for all databases, registers and websites, including any filters and limits used.	Page 3, Supplementary Table 2
Selection process	8	Specify the methods used to decide whether a study met the inclusion criteria of the review, including how many reviewers screened each record and each report retrieved, whether they worked independently, and if applicable, details of automation tools used in the process.	Page 3
Data collection process	9	Specify the methods used to collect data from reports, including how many reviewers collected data from each report, whether they worked independently, any processes for obtaining or confirming data from study investigators, and if applicable, details of automation tools used in the process.	Page 3
Data items	10a	List and define all outcomes for which data were sought. Specify whether all results that were compatible with each outcome domain in each study were sought (e.g. for all measures, time points, analyses), and if not, the methods used to decide which results to collect.	Page 3
	10b	List and define all other variables for which data were sought (e.g. participant and intervention characteristics, funding sources). Describe any assumptions made about any missing or unclear information.	Page 3 Supplementary Tables 5,10
Study risk of bias assessment	11	Specify the methods used to assess risk of bias in the included studies, including details of the tool(s) used, how many reviewers assessed each study and whether they worked independently, and if applicable, details of automation tools used in the process.	Page 3,4
Effect	12	Specify for each outcome the effect measure(s) (e.g. risk ratio, mean difference) used in the synthesis or presentation of	Page 4

Section and Topic	Item #	Checklist item	Location where item is reported
measures		results.	
Synthesis methods	13a	Describe the processes used to decide which studies were eligible for each synthesis (e.g. tabulating the study intervention characteristics and comparing against the planned groups for each synthesis (item #5)).	Page 4
	13b	Describe any methods required to prepare the data for presentation or synthesis, such as handling of missing summary statistics, or data conversions.	Page 4
	13c	Describe any methods used to tabulate or visually display results of individual studies and syntheses.	Page 4
	13d	Describe any methods used to synthesize results and provide a rationale for the choice(s). If meta-analysis was performed, describe the model(s), method(s) to identify the presence and extent of statistical heterogeneity, and software package(s) used.	Page 4
	13e	Describe any methods used to explore possible causes of heterogeneity among study results (e.g. subgroup analysis, meta-regression).	Page 4
	13f	Describe any sensitivity analyses conducted to assess robustness of the synthesized results.	Page 4
Reporting bias assessment	14	Describe any methods used to assess risk of bias due to missing results in a synthesis (arising from reporting biases).	Page 4
Certainty assessment	15	Describe any methods used to assess certainty (or confidence) in the body of evidence for an outcome.	Page 4
**RESULTS**			
Study selection	16a	Describe the results of the search and selection process, from the number of records identified in the search to the number of studies included in the review, ideally using a flow diagram.	Page 4
	16b	Cite studies that might appear to meet the inclusion criteria, but which were excluded, and explain why they were excluded.	Page 4,8
Study characteristics	17	Cite each included study and present its characteristics.	Page 4,8
Risk of bias in studies	18	Present assessments of risk of bias for each included study.	Supplementary Table 8, Supplementary Figure 6
Results of individual studies	19	For all outcomes, present, for each study: (a) summary statistics for each group (where appropriate) and (b) an effect estimate and its precision (e.g. confidence/credible interval), ideally using structured tables or plots.	Table 1, 2<br>Figure 1, 2
Results of syntheses	20a	For each synthesis, briefly summarise the characteristics and risk of bias among contributing studies.	Page 8,12
	20b	Present results of all statistical syntheses conducted. If meta-analysis was done, present for each the summary estimate and its precision (e.g. confidence/credible interval) and measures of statistical heterogeneity. If comparing groups, describe the direction of the effect.	Figure 1, 2
	20c	Present results of all investigations of possible causes of heterogeneity among study results.	Page 9,12
	20d	Present results of all sensitivity analyses conducted to assess the robustness of the synthesized results.	Page 8,12
Reporting biases	21	Present assessments of risk of bias due to missing results (arising from reporting biases) for each synthesis assessed.	Page 13

Section and Topic	Item #	Checklist item	Location where item is reported
Certainty of evidence	22	Present assessments of certainty (or confidence) in the body of evidence for each outcome assessed.	Page 13
**DISCUSSION**			
Discussion	23a	Provide a general interpretation of the results in the context of other evidence.	Page 13
	23b	Discuss any limitations of the evidence included in the review.	Page 14
	23c	Discuss any limitations of the review processes used.	Page 14
	23d	Discuss implications of the results for practice, policy, and future research.	Page 14
**OTHER INFORMATION**			
Registration and protocol	24a	Provide registration information for the review, including register name and registration number, or state that the review was not registered.	Page 3
	24b	Indicate where the review protocol can be accessed, or state that a protocol was not prepared.	Page 3
	24c	Describe and explain any amendments to information provided at registration or in the protocol.	NA
Support	25	Describe sources of financial or non-financial support for the review, and the role of the funders or sponsors in the review.	Page 15
Competing interests	26	Declare any competing interests of review authors.	Page 15
Availability of data, code and other materials	27	Report which of the following are publicly available and where they can be found: template data collection forms; data extracted from included studies; data used for all analyses; analytic code; any other materials used in the review.	Page 15

ESM Table 2. Search strategy.

	MEDLINE		*EMBASE*		*The Cochrane library*
	**Through March 9th, 2021**		**Through March 9th, 2021**		**Through March 9th, 2021**
1	Diet, Scandinavian/	1	Diet, Scandinavian/	1	(Scandinavian adj3 diet).mp.
2	(Scandinavian adj3 diet).mp.	2	(Scandinavian adj3 diet).mp.	2	Scandinavian diet*.mp.
3	Scandinavian diet*.mp.	3	Scandinavian diet*.mp.	3	(Baltic sea adj3 diet).mp.
4	Diet, Baltic sea/	4	Diet, Baltic sea/	4	Baltic sea diet*.mp.
5	(Baltic sea adj3 diet).mp.	5	(Baltic sea adj3 diet).mp.	5	(Finnish adj3 diet).mp.
6	Baltic sea diet*.mp.	6	Baltic sea diet*.mp.	6	Finnish diet*.mp.
7	Diet, Finnish/	7	Diet, Finnish/	7	(Danish adj3 diet).mp.
8	(Finnish adj3 diet).mp.	8	(Finnish adj3 diet).mp.	8	Danish diet*.mp.
9	Finnish diet*.mp.	9	Finnish diet*.mp.	9	(Swedish adj3 diet).mp.
10	Diet, Danish/	10	Diet, Danish/	10	Swedish diet*.mp.
11	(Danish adj3 diet).mp.	11	(Danish adj3 diet).mp.	11	(Icelandic adj3 diet).mp.
12	Danish diet*.mp.	12	Danish diet*.mp.	12	Icelandic diet*.mp.
13	Diet, Swedish/	13	Diet, Swedish/	13	(Nordic adj3 diet).mp.
14	(Swedish adj3 diet).mp.	14	(Swedish adj3 diet).mp.	14	Nordic diet*.mp.
15	Swedish diet*.mp.	15	Swedish diet*.mp.	15	(Malmo adj3 diet).mp.
16	Diet, Icelandic/	16	Diet, Icelandic/	16	Malmo diet*.mp.
17	(Icelandic adj3 diet).mp	17	(Icelandic adj3 diet).mp.	17	(Sami adj3 diet).mp.
18	Icelandic diet*.mp.	18	Icelandic diet*.mp.	18	Sami diet*.mp.
19	Diet, Nordic/	19	Diet, Nordic/	19	(Norwegian adj3 diet).mp.
20	(Nordic adj3 diet).mp.	20	(Nordic adj3 diet).mp.	20	Norwegian diet*.mp.
21	Nordic diet*.mp.	21	Nordic diet*.mp.	21	(Faroese islands adj3 diet).mp.
22	Diet, Malmo /	22	Diet, Malmo /	22	Faroese islands diet*.mp.
23	(Malmo adj3 diet).mp.	23	(Malmo adj3 diet).mp.	23	or/1-22
24	Malmo diet*.mp.	24	Malmo diet*.mp.	24	Stroke/
25	Diet, Faroese islands/	25	Diet, Faroese islands/	25	stroke.mp.

26	(Faroese islands adj3 diet).mp.	26	(Faroese islands adj3 diet).mp.	26	cerebrovascular accident.mp.
27	Faroese islands diet*.mp.	27	Faroese islands diet*.mp.	27	(fatal adj3 stroke).mp.
28	(Sami adj3 diet).mp.	28	(Sami adj3 diet).mp.	28	Cerebral Hemorrhage/
29	Sami diet*.mp.	29	Sami diet*.mp.	29	hemorrhagic stroke.mp.
30	Diet, Sami/	30	Diet, Sami/	30	Intracranial Hemorrhages/
31	(Norwegian adj3 diet).mp.	31	(Norwegian adj3 diet).mp.	31	Brain Ischemia/
32	Norwegian diet*.mp.	32	Norwegian diet*.mp.	32	brain ischemia.mp.
33	Diet, Norwegian/	33	Diet, Norwegian/	33	Cerebral Infarction/
34	or/1-33	34	or/1-33	34	Peripheral Arterial Disease/
35	exp Stroke/	35	exp cerebrovascular accident/	35	peripheral arterial disease.mp.
36	(fatal adj3 stroke).mp.	36	stroke.mp.	36	Heart Failure/
37	non fatal stroke.mp.	37	(fatal adj3 stroke).mp.	37	Myocardial Ischemia/
38	hemorrhagic stroke.mp.	38	non fatal stroke.mp.	38	myocardial ischemia.mp.
39	exp Intracranial Hemorrhages/	39	hemorrhagic stroke.mp.	39	Myocardial Infarction/
40	exp Intracranial arterial diseases/	40	exp brain hemorrhage/	40	myocardial infarction.mp.
41	ischemic stroke.mp.	41	intracranial hemorrhage.mp.	41	cardiovascular disease mortality.mp.
42	exp Brain Ischemia/	42	exp cerebral artery disease/	42	cardiovascular disease death.mp.
43	exp Cerebral Infarction/	43	intracranial arterial disease.mp.	43	CVD mortality.mp.
44	exp Peripheral Arterial Disease/	44	ischemic stroke.mp.	44	Cardiovascular Diseases/
45	peripheral artery disease.mp.	45	exp brain ischemia/	45	cardiovascular disease.mp.
46	exp heart failure/	46	exp brain infarction/	46	CVD.mp.
47	heart failure.mp.	47	exp peripheral occlusive artery disease/	47	Coronary Disease/
48	exp myocardial ischemia/	48	peripheral artery disease.mp.	48	coronary disease.mp.
49	exp myocardial infarction/	49	exp heart failure/	49	cerebrovascular.mp.
50	cardiovascular disease mortality.mp.	50	heart failure.mp.	50	OGTT.mp.
51	cardiovascular disease	51	exp heart muscle	51	oral glucose tolerance

	death.mp.		ischemia/		test'.mp.
52	CVD death.mp.	52	exp heart infarction/	52	exp Hemoglobin A, Glycosylated/
53	CVD mortality.mp.	53	cardiovascular disease mortality.mp.	53	hba1c.mp.
54	cardiovascular disease.mp.	54	cardiovascular disease death.mp.	54	insulin*.mp.
55	exp cardiovascular disease/	55	CVD mortality.mp.	55	glycemia.mp.
56	CVD.mp.	56	CVD death.mp.	56	exp Glucose/
57	coronary disease.mp.	57	cardiovascular disease.mp.	57	exp Hyperglycemia/
58	exp Coronary Disease/	58	exp cardiovascular disease/	58	hyperinsulin*.mp.
59	cerebrovascular.mp.	59	CVD.mp.	59	dysglycemia.mp.
60	cerebral vascular.mp.	60	coronary disease.mp.	60	exp diabetes mellitus/
61	OGTT.mp.	61	exp coronary artery disease/	61	metabolic syndrome.mp.
62	exp Hemoglobin A, Glycosylated /	62	cerebrovascular.mp.	62	exp Body Weight/
63	hba1c.mp.	63	cerebral vascular.mp.	63	body weight*.mp.
64	insulin*.mp.	64	exp oral glucose tolerance test/	64	exp Body Mass Index/
65	glycemia.mp.	65	OGTT.mp.	65	body mass index.mp.
66	exp Glucose/	66	exp hemoglobin A1c/	66	BMI.mp.
67	exp Hyperglycemia/	67	hba1c.mp.	67	exp Waist Circumference/
68	hyperinsulin*.mp.	68	insulin*.mp.	68	waist circumference.mp.
69	dysglycemia.mp.	69	exp glucose blood level/	69	exp overweight/
70	exp diabetes mellitus/	70	glycemia.mp.	70	overweight.mp.
71	metabolic syndrome.mp.	71	exp glucose/	71	exp Obesity/
72	exp Body Weight/	72	'impaired fasting glucose'.mp.	72	exp Obesity, Abdominal/
73	body weight*.tw.	73	hyperglycemia.mp.	73	exp Obesity, Morbid/
74	exp Body Mass Index/	74	'impaired glucose tolerance'.mp.	74	obesity.mp.
75	body mass index.tw.	75	hyperinsulin*.mp.	75	body fat.mp.
76	BMI.tw.	76	dysglycemia.mp.	76	hypertension.mp.
77	exp Waist	77	exp diabetes	77	blood

	Circumference/		mellitus/		pressure.mp.
78	waist circumference.tw.	78	exp insulin dependent diabetes mellitus/	78	systolic blood pressure.mp.
79	exp overweight/	79	exp non insulin dependent diabetes mellitus/	79	diastolic blood pressure.mp.
80	overweight.tw.	80	exp pregnancy diabetes mellitus/	80	hypertension.mp.
81	exp Obesity/	81	exp metabolic syndrome X/	81	SBP.mp.
82	exp Obesity, Abdominal/	82	exp Body Weight/	82	DBP.mp.
83	exp Obesity, Morbid/	83	body weight*.tw.	83	exp lipoproteins/ or exp cholesterol/ or exp hyperlipidemias/ or (lipid or lipids).mp.
84	obesity.tw.	84	exp Body Mass Index/	84	(cholesterol or cholesterols).mp.
85	body fat.tw.	85	body mass index.tw.	85	hdl.mp.
86	exp Hypertension/	86	BMI.tw.	86	("high density lipoprotein" or "high density lipoproteins").mp.
87	Blood Pressure/	87	exp Waist Circumference/	87	ldl.mp.
88	"diastolic blood pressure".mp.	88	waist circumference.tw.	88	("low density lipoprotein" or "low density lipoproteins").mp.
89	"systolic blood pressure".mp.	89	exp Overweight/	89	(hyperlipemia* or hyperlipaemia*).mp.
90	hypertension.mp.	90	overweight.tw.	90	(hyperlipidemia* or hyperlipidaemia*).mp.
91	SBP.mp.	91	exp Obesity/	91	(lipidemia* or lipidaemia*).mp.
92	DBP.mp.	92	exp Obesity, Abdominal/	92	(lipemia* or lipaemia*).mp.
93	exp lipoproteins/ or exp cholesterol/ or exp hyperlipidemias/ or (lipid or lipids).mp.	93	Obesity, Morbid/	93	(lipemic or lipaemic).mp.

94	(cholesterol or cholesterols).mp.	94	obesity.tw.	94	triglycerides.mp.
95	hdl.mp.	95	body fat.tw.	95	hypertriglyceridemia.mp.
96	("high density lipoprotein" or "high density lipoproteins").mp.	96	exp Hypertension/	96	TG.mp.
97	ldl.mp.	97	exp Blood Pressure/	97	triacylglycerol*.mp.
98	("low density lipoprotein" or "low density lipoproteins").mp.	98	"systolic blood pressure".mp.	98	TAG.mp.
99	(hyperlipemia* or hyperlipaemia*).mp.	99	"diastolic blood pressure".mp.	99	dyslipidemia.mp.
100	(hyperlipidemia* or hyperlipidaemia*).mp.	100	SBP.mp.	100	Inflamm*.mp
101	(lipidemia* or lipidaemia*).mp.	101	DBP.mp.	101	C-reactive protein.mp
102	(lipemia* or lipaemia*).mp.	102	(cholesterol or cholesterols).mp.	102	CRP.mp
103	(lipemic or lipaemic).mp.	103	hdl.mp.	103	or/24-103
104	exp Triglycerides/	104	exp lipoproteins/ or exp cholesterol/ or exp hyperlipidemias/ or (lipid or lipids).mp.		
105	triglyceride*.mp.	105	("high density lipoprotein" or "high density lipoproteins").mp.		
106	hypertriglyceridemia*.mp.	106	ldl.mp.		
107	exp Hypertriglyceridemia/	107	("low density lipoprotein" or "low density lipoproteins").mp.		
108	exp Dyslipidemias/	108	(hyperlipemia* or hyperlipaemia*).mp.		
109	triacylglycerol*.mp.	109	(hyperlipidemia* or hyperlipidaemia*).mp.		

110	dyslipidaemia*.mp.	110	(lipidemia* or lipidaemia*).mp.
111	dyslipidemia.mp.	111	(lipemia* or lipaemia*).mp.
112	Inflamm*.mp	112	(lipemic or lipaemic).mp.
113	C-reactive protein.mp	113	exp Triglycerides/
114	CRP.mp	114	exp Hypertriglyceridemia/
115	or/35-114	115	hypertriglyceridemia*.mp.
116	exp cohort studies/	116	triglyceride*.mp.
117	cohort$.tw.	117	triacylglycerol*.mp.
118	controlled clinical trial.pt.	118	dyslipidemia*.mp.
119	epidemiologic methods/	119	dyslipidaemia*.mp.
120	limit 35 to yr=1971-1988	120	exp Dyslipidemias/
121	116 or 117 or 118 or 120	121	Inflamm*.mp
122	34 and 115 and 121	122	C-reactive protein.mp
123	"randomized controlled trial".pt.	123	CRP.mp
124	(random$ or placebo$ or single blind$ or double blind$ or triple blind$).ti,ab.	124	or/35-123
125	(retraction of publication or retracted publication).pt.	125	exp cohort analysis/
126	123 or 124 or 125	126	exp longitudinal study/
127	(animals not humans).sh.	127	exp prospective study/
128	((comment or editorial or meta-analysis or practice-guideline or review or letter or journal correspondence) not "randomized controlled trial").pt.	128	exp follow up/
129	(random sampl$ or random digit$ or random	129	cohort$.tw.

	effect$ or random survey or random regression).ti, ab. not "randomized controlled trial".pt.			Original search date: August 1st 2019; update search date: March 9th, 2021.
130	126 not (127 or 128 or 129)	130	125 or 126 or 127 or 128 or 129	
131	34 and 115 and 128	131	34 and 123 and 130	
132	121 or 131	132	(random$ or placebo$ or single blind$ or double blind$ or triple blind$).ti,ab.	
		133	RETRACTED ARTICLE/	
		134	131 or 133	
		135	(animal$ not human$).sh,hw.	
		136	(book or conference paper or editorial or letter or review).pt. not exp randomized controlled trial/	
		137	(random sampl$ or random digit$ or random effect$ or random survey or random regression).ti, ab. not exp randomized controlled trial/	
		138	133 not (135 or 136 or 137)	
		139	34 and 123 and 138	
		140	131 or 139	

ESM Table 3. Eligibility criteria for prospective cohort studies

Participants	Inclusion criteria	Exclusion criteria	Outcome
All individuals, both children, and adults, regardless of health status.	• Prospective cohort studies<br>• Duration >= 1 year<br>• Assessment of the exposure of a Nordic Diet<br>• Ascertainment of viable data by level of exposure	• Ecological, cross-sectional, retrospective observational studies, clinical trials, and non-human studies<br>• Duration < 1 year<br>• non assessment of exposure of a Nordic diet<br>• No ascertainment viable clinical outcome data by level of exposure	Cardiovascular Diseases<br>Coronary Heart Disease<br>Stroke<br>Mortality<br>Diabetes

ESM Table 4. PICOTS[a] framework for inclusion of randomized controlled trials

Participants	Intervention	Comparison	Outcome	Time	Study Design
All individuals, both children, and adults, regardless of health status.	Nordic diets intervention	Habitual or usual or western diet	Adiposity, glycemic control, established blood lipid targets, blood pressure, inflammation	≥ 3 weeks	Human randomized controlled trials

a Population, Intervention, Comparator, Outcome, Time, and Study design

ESM Table 5. Characteristics of included cohorts.

Study, year	Cohort	Sex	Population*	Country	Ethnicity	N	Cases	Age	Follow-up (years)	Mean Follow-up (years)	Method of Measurement of Exposure	Quantile divisions (score division)	Nordic diet index	Outcome	Funding Sources
**Gunge et al. 2017**	**Danish Diet, Cancer and Health cohort**	M	Free of cancer	Denmark	Caucasian	25,759	1,669	50-64[a]	1993-2009	13.6	192-item SFFQ	Category, (0,1,2,3,4,5, 6)	HNFI	CHD incidence	A
		F				28,809	653								
**Warensjo Lemming et al. 2018**	**Swedish Mammography Cohort**	F	General	Sweden	Caucasian	33,341	3003	61[b]	1997-2014	17	96-item FFQ	Tertiles (0–1, 2-4,5-6)	HNFI	CVD mortality	A
**Tertsunen et al 2020**	**Kuopio Ischaemic Heart Disease Risk Factor Study**	M	General	Finland	Caucasian	1547	250	42-60[a]	1984–1989, 2014	23.6[a]	Dietary records 4-day	Tertiles (0–1, 2-4,5-6)	Modified Baltic Sea Diet Score	CVD mortality	A
**Lacoppidan et al 2015**	**Danish Diet, Cancer and Health cohort**	M	Free of cancer	Denmark	Caucasian	26,107	4097	50-64[a]	1993-1997,2011	15.3[a]	192-item SFFQ	Category, (0,1,2,3,4,5, 6)	HNFI	T2DM	A
		F				28,953	3269								
**Ewers et al 2020**	**The Copenhagen General Population Study**	M + F	General	Denmark	Caucasian	88,818	2982	58[b]	2003-2015,2018	9.2	Short FFQ	Quantiles (Very high/High, Intermediate, Very Low/Low)	Danish food-based dietary guidelines	CVD mortality	A
**Lassale et al. 2016**	**EPIC**	M + F	Free of cancer and diabetes	Denmark, France, Germany, Greece, Italy, the Netherlands, Norway, Spain, Sweden, and the United Kingdom	Caucasian	451,256	3761	25-70[a]	Recruit 1992 - 2000	12.8	Dietary questionnaires (validation with 24h recalls, FFQ, dietary records)	Quantiles (0,1,2,3,4,5, 6)	HNFI	CVD mortality	A
**Drake et al. 2013**	**Malmö Diet and Cancer cohort**	M	Free of diabetes	Sweden	Caucasian	6940	444	45–73[a]	Recruit 1991-1996, 2008	14.2[a]	7-d food diary, 168-item FFQ, diet history interview	Tertiles (0-1, 2-3,4-6)	DQI-SNR	CVD mortality	A
		F				10,186	265	44–73[a]							
**Hansen et al. 2017**	**Danish Diet, Cancer and Health cohort**	M + F	Free of cancer	Denmark	Caucasian	55,338	2283	56.1[b]	Recruit 1993-1997	13.5	192-tem SFFQ	Tertiles (0-1, 2-3,4-6)	HNFI	Stroke incidence	A
**Hlebowicz et al. 2013**	**Malmö Diet and Cancer cohort**	M	Free of diabetes	Sweden	Caucasian	6940	1093	45–73[a]	1991-2008	14[a]	7-d food diary, 168-item FFQ, diet history interview	Tertiles (0-1, 2-3,4-6)	DQI-SNR	CVD incidence	A
		F				10,186	703	44–74[a]							
**Roswall et al. 2015**	**Swedish Women's Lifestyle and Health Cohort**	F	General	Sweden	Caucasian	43,310	8383	29–49[a]	1991–1992,2012	21.3	80-item FFQ, 7-day records in 129 women	Tertiles (0-1, 2-3,4-6)	HNFI	CVD incidence	A
							698							Stroke incidence	

							1019							CHD incidence	
**Roswall et al. 2015**	**Swedish Women's Lifestyle and Health Cohort**	F	General	Sweden	Caucasian	44,961	270	29–49[a]	1991–1992,2012	21.3	80-item FFQ, 7-day records in 129 women	Tertiles (0-1, 2-3,4-6)	HNFI	CVD mortality	A
**Kanerva et al 2014**	**Helsinki Birth Cohort Study**	M + F	Free of diabetes	Denmark	Caucasian	6744	541	47-62[a]	2000-2010	9.4[a]	128-item FFQ	Quantiles (0-25)	Baltic Sea Diet Score	T2DM	A+I
	**Health 2000 Survey**									11.3[a]					
**Mandalazi et al 2016**	**Malmö Diet and Cancer cohort**	M	Free of diabetes	Sweden	Caucasian	26,868	1,859	44-74[a]	1991-1996,2014	17[a]	7-d food diary, 168-item FFQ, diet history interview	Tertiles (0-1, 2-4,5-6)	DQI-SNR	T2DM	A
		F					1,979								
**Galbete et al. 2018**	**EPIC-Potsdam**	M + F	Free of cancer and diabetes	Germany	Caucasian	23,485	312	35-65[a]	1994-1998, 2009	10.8[a]	148-item FFQ	Tertiles (0-7, 8-10,11-18)	Nordic diet score	CHD incidence	A
							321							Stroke incidence	
														T2DM	
**Puaschitz et al. 2019**	**Western Norway B-vitamin Intervention Trial (WENBIT)**	M + F	Stable angina	Norway	Caucasian	2019	171	28-85[a]	1999-2004, 2010	10.5[a]	169-item FFQ	Tertiles (0-1, 2-3,4-6)	HNFI	CVD mortality	A
							307		1999-2004, 2013	7.5[a]				CHD incidence	

Abbreviations: A, Agency; M, males; F, females; NA, not available; SFFQ, Short Food Frequency Questionnaire; HNFI, Healthy Nordic Food Index; I, Industry; MI, myocardial infraction; T2DM, Type 2 diabetes melitus; FFQ, food frequency questionnaire; DQI-SNR, diet quality index (DQI) hat assesses adherence to the 2005 Swedish Nutrition Recommendations (SNR); IHD, ischemic heart disease.

[a] Age range; [b]Median value given *Population excludes individuals with CVD at baseline

ESM Table 6. Dietary scores used to assess adherence to the Nordic dietary pattern.

Studies	Nordic Diet Index	Scoring Categories	Primary Food Components	Cut-offs	Reference Guidelines
**[76, 77, 79-81, 83, 85, 88]**	Healthy Nordic food index	0 - 6 (low adherence – high adherence)	Fish, cabbage, root vegetables, rye bread, oatmeal, apples, pears	Population based	A priory chosen food items due to expected beneficial health effects
**[78,84, 89]**	Diet quality index (DQI) that assesses adherence to the Swedish nutrition recommendations (SNR) and the Swedish dietary guidelines (SDG)<br><br>(DQI-SNR)	0-6 (0 or 1 low, 2 or 3 medium, 4-6 high score)	SFA*, PUFA*, fish and shellfish dietary fiber, fruit and vegetables, and sucrose.<br><br>**SFA and PUFA as indicators of fat intake*	Serving based	Swedish nutrition recommendations, Swedish dietary guidelines
**[82]**	Nordic diet score	0-18 (0-7, 8-10,11-18 low adherence – high adherence)	Fish, cabbage and cruciferous vegetables, root vegetables, potatoes, whole grain and rye bread, berries, apples, pears, low-fat dairy products, vegetable fats (excluding olive oil)	Population based	Healthy Nordic Food Index, New Nordic Diet, The Baltic Sea diet score
**[87]**	Baltic Sea Diet Score	Population-based consumption quartiles or medians as cut-offs	Berries, apples, pears, tomato, cucumber, cabbage, roots, peas, lettuce, rye, oats, barley, fat-free milk and milk < 2% fat, salmon, freshwater fish, beef, pork, processed meat products, sausages, total fat as a percentage of total energy intake<br>Ratio of PUFA to SFA + trans-fatty acids<br>Ethanol	Population based	Baltic Sea Diet Pyramid, Nordic multicenter SYSDIET study

**[90]**	Modified Baltic Sea Diet Score	2-25<br>(2–10, 11–12, 13–15, 16–25 low adherence – high adherence)	All fruits, berries, roots, pulses, vegetables, whole grains, fat-free milk and milk < 2% fat, salmon, freshwater fish, processed and unprocessed meat, total fat as a percentage of total energy intake<br>Ratio of PUFA to SFA + trans-fatty acids<br>Ethanol	Population based	Baltic Sea Diet Score
**[86]**	Danish food-based dietary guidelines	Q1-Q5<br>(Very high-Q1, high, intermediate, low, very low adherence-Q5)	High intakes of unsaturated fats, vegetables, fruits, fish<br><br>Low intakes for sugar sweetened beverages, cold meat cuts and fast food.	Serving based	Danish food-based dietary guidelines

ESM Table 7. Confounding variables of included cohorts.

Study	Gunge et al. 2017 Danish Diet, Cancer and Health cohort	Warensjo Lemming et al. 2018 Swedish Mammography Cohort	Lassale et al. 2016 EPIC	Drake et al. 2013 Malmö Diet and Cancer cohort	Hansen et al. 2017 Danish Diet, Cancer and Health cohort	Puaschitz et al. 2019 Western Norway B-vitamin Intervention Trial	Hlebowicz et al. 2013 Malmö Diet and Cancer cohort	Roswall et al. 2015 Swedish Women's Lifestyle and Health Cohort [93]
**Number of variables in fully adjusted model**	17	7	7	12	12	9	8	11
**Number of multivariable models presented**	4	2	2	2	3	3	2	4
**Timing of measurement of confounding variables**	Baseline	Baseline	Baseline	Baseline	Baseline	Baseline	Baseline	Baseline
**Pre-specified primary confounding variables**								
Age	✓	✓	✓	✓	✓	✓	✓	✓
**Pre-specified secondary confounding variables**								
Sex	✓	✓	✓	✓	✓	✓	✓	✓
Body mass index, weight	✓	✓	✓	✓	✓			✓
Waist circumference	✓			✓	✓		✓	
Family history of CVD								
Energy Intake	✓	✓		✓		✓		✓
Smoking	✓	✓	✓	✓	✓	✓	✓	✓
Exercise/physical activity	✓	✓	✓	✓	✓	✓	✓	
Diabetes/Dysglycemia					✓	✓		
Dyslipidemia	✓				✓			
Hypertension/SBP	✓				✓	✓	✓	
**Other confounding variables**								
Education	✓	✓	✓	✓	✓		✓	✓
Alcohol				✓	✓	✓	✓	✓
Alcohol from wine	✓							
Alcohol from beer/spirits	✓							
Total Cholesterol	✓				✓			
Non-fermented milk		✓						
Meat, red meat	✓							✓
Hormonal replacement therapy	✓							
Menopause	✓							
Method of assessment				✓			✓	
Cohabiting status		✓		✓			✓	
Processed meat consumption	✓							✓
Tobacco consumption								✓
Time since cessation of smoking	✓							✓
Charlson's comorbidity index		✓						
Other	Time under study	Diet score, non-fermented milk	Study centre	Season	Atrial fibralation	Statin use	Economic status, Season	

ESM Table 7. Confounding variables of included cohorts (continued).

Study	Mandalazi et al. 2016 Malmö Diet and Cancer cohort	Karneva et al. 2014 Helsinki Birth Cohort Study, Health 2000 Survey	Ewers et al. 2020 the Copenhagen General Population Study	Lacoppidan et al. 2015 Danish Diet, Cancer and Health cohort	Tertsunen et al. 2020 Kuopio Ischaemic Heart Disease Risk Factor Study	Roswall et al. 2015 Swedish Women's Lifestyle and Health Cohort [92]	Galbete et al. 2018 EPIC-Potsdam
**Number of variables in fully adjusted model**	10	8	11	9	9	12	12
**Number of multivariable models presented**	5	3	3	4	2	4	2
**Timing of measurement of confounding variables**	Baseline	Baseline	Baseline	Baseline	Baseline	Baseline	Baseline
**Pre-specified primary confounding variables**							
Age	✓	✓	✓	✓	✓	✓	✓
**Pre-specified secondary confounding variables**							
Sex	✓	✓	✓	✓	✓	✓	✓
Body mass index, weight	✓		✓	✓		✓	
Waist circumference	✓						
Family history of CVD							
Energy Intake		✓		✓	✓	✓	✓
Smoking	✓	✓	✓	✓	✓	✓	✓
Exercise/physical activity	✓	✓	✓	✓	✓		✓
Diabetes/Dysglycemia			✓			✓	
Dyslipidemia	✓						
Hypertension/SBP	✓		✓			✓	✓
**Other confounding variables**							
Education	✓	✓	✓	✓	✓	✓	✓
Alcohol	✓		✓	✓		✓	✓
Alcohol from wine							
Alcohol from beer/spirits							
Total Cholesterol							
Non-fermented milk							
Meat, red meat				✓			
Hormonal replacement therapy							
Menopause							
Method of assessment							
Cohabiting status							
Processed meat consumption						✓	
Tobacco consumption						✓	
Time since cessation of smoking						✓	
Charlson's comorbidity index							
Other	season, method of dietary assesment	Abdominal obesity, vitamin D intake	LDL-Cholesterol, Income		Income, marital status, examination year		Multivitamin

ESM Table 8. Newcastle-Ottawa Scale (NOS) scores of included cohorts.

Study	Selection[a]	Outcome[b]	Comparability[c]	Total[d]
**The Danish Diet, Cancer and Health cohort [76]**	3	3	1	7
**Swedish Mammography Cohort [81]**	2	3	1	6
**EPIC [85]**	3	3	1	7
**Malmö Diet and Cancer cohort [84]**	4	3	1	8
**The Danish Diet, Cancer and Health cohort [77]**	3	3	2	8
**Western Norway B-vitamin Intervention Trial [83]**	3	3	1	7
**EPIC-Potsdam [82]**	3	3	1	7
**Malmö Diet and Cancer cohort [78]**	4	3	1	8
**Swedish Women's Lifestyle and Health Cohort [83]**	2	3	1	6
**Swedish Women's Lifestyle and Health Cohort [82]**	3	3	1	7
**Malmö Diet and Cancer cohort [89]**	3	4	1	8
**Helsinki Birth Cohort Study, Health 2000 Survey [87]**	3	4	1	8
**The Copenhagen General Population Study [86]**	3	3	2	8
**The Danish Diet, Cancer and Health Cohort Study [88]**	3	3	2	8
**Kuopio Ischaemic Heart Disease Risk Factor Study [90]**	3	3	2	8

Abbreviations: EPIC=European Prospective Investigation into Cancer and Nutrition

[a]Maximum 4 points awarded for cohort representativeness, selection of non-exposed cohort, exposure assessment, and demonstration outcome not present at baseline

[b]Maximum 3 points awarded for follow-up length, adequacy of follow-up, and outcome assessment

[c]Maximum 2 points awarded for controlling for the pre-specified primary confounding variable (age) and 4 of the 5 secondary (markers of overweight/obesity, family history of diabetes, energy intake, physical activity, sex) confounding variables

[d]A maximum of 9 points could be awarded. Cohorts with NOS ≥6 are considered high quality.

ESM Table 9. Selected sensitivity analyses in which the systematic removal of a cohort study altered the significance of the effect estimate or the evidence for heterogeneity.

Outcome	Removal of	MD [95% CI], P_{MD}<br>I^2, P_Q
**CVD INCIDENCE**	Roswall et al. 2015 [83][b]	0.70 [0.61, 0.81], P_{MD}<0.001,<br>I^2=0%, P_Q=0.63
	Hlebowicz et al. 2013 - Males [c]	0.96 [0.90, 1.02], P_{MD}=0.16<br>I^2=85%, P_Q=0.01
	Hlebowicz et al. 2013 - Females[c]	0.95 [0.89, 1.01], P_{MD}=0.08<br>I^2=92%, P_Q<0.001
**T2DM INCIDENCE**	Lacoppidan et al. 2015 – Males[b]	1.01 [0.93, 1.09], P_{MD}=0.89<br>I^2=0%, P_Q=0.59
**CHD INCIDENCE**	Roswall et al. 2015 [83][a]	0.82 [0.68, 0.97], P_{MD}=0.02<br>I^2=27%, P_Q=0.25
	Gunge et al. 2017 [b]	0.96 [95% CI 0.85, 1.09], P_{MD}=0.51, I^2=7%,<br>P_Q=0.36.
**STROKE INCIDENCE**	Hansen et al. 2017 [c]	0.86 [0.82, 1.17], P_{MD}=0.80<br>I^2=0%, P_Q=0.96
	Galbete et al. 2018 [c]	0.88 [0.75, 1.03], P=0.10<br>I^2=36%, P_Q=0.21

P_{MD}, mean difference p-value, P_Q, Cochrane Q p-value, T2DM, Type 2 Diabetes Mellitus.
[a] removal of this study results in significance of the overall effect
[b] removal of this study explains heterogeneity
[c] removal of this study results in a loss of significance of the overall effect

ESM Table 10. Characteristics of included RCTs.

Study, Year	Intervention, Control	Participants (M, W)	Mean age, y (SD or range)	Baseline BMI (kg/m^2), mean (SD)	Baseline LDL-C (mmol/L), mean (SD)	Setting	Design	Feeding Control[a]	Intervention or Comparator	Diet (% C:F:P)[b]	Energy Balance[c]	Outcome	Follow-up duration, weeks	Funding Sources[e]
Gotfredsen et al. 2020	Intervention	72 individuals with IHD Risk Factors (29M, 43W)	51.8 (9.8)	26.9 (3.6)	3.10 (0.91)	Denmark	Parallel	DA	Official dietary guidelines	Not available	Neutral	HbA1c, Glucose, Insulin, LDL-C, HDL-C,**Non-HDL-C, TG, BW, BMI WC, SBP, DBP, CRP	24	A
	Control	73 individuals with IHD Risk Factors (30M, 43W)	49.2 (9.8)	26.5 (3.9)	3.24 (0.76)				Habitual diet	Not available				
Poulsen et al. 2014	Intervention	91 OB	42.7 (13.1)*	30.1 (4.6)*	2.95 (0.84)*	Denmark	Parallel	Suppl	New Nordic Diet	52:30:18	Neutral	Glucose, Insulin, LDL-C, HDL-C, TG, BW, WC, SBP, DBP, CRP	26	A, I
	Control	56 OB	41.0 (13.0)*	30.5 (5.3)*	2.96 (0.81)*				Average Danish Diet	50:35:15				
Uusitupa et al. 2013	Intervention	96 MetS (~29M, 67W)	54.0 (8.5)*	31.6 (3.5)*	3.25 (0.80)	Nordic Countries[†]	Parallel	Suppl, DA	Healthy Nordic Diet	45-52:30-35:18-20	Neutral	Glucose, LDL-C, Non-HDL-C, HDL-C TG, ApoB BW, SBP, DBP, CRP	18 (24-wk for 2 sites)	A, I
	Control	70 MetS (~21M, 49W)	54.9 (8.6)*	31.7 (2.8)*	3.21 (0.89)				Usual Nordic diet	45-47:35:18-20				
Adamsson et al. 2010	Intervention	44 mildly HC (17M, 27W)	52.6 (7.8)	26.3 (3.2)	4.0 (0.6)	Sweden	Parallel	Suppl to ND only	Healthy Nordic Diet	45-60:25-35:10-20	Neutral	Glucose, Insulin, LDL-C, HDL-C,**Non-HDL-C, TG, ApoB BW, BMI SBP, DBP, CRP	6	A
	Control	42 mildly HC (15M, 27W)	53.4 (8.1)	26.5 (3.3)	4.2 (1.0)				Usual Western diet	NR				
Huseinovic et al.2016	Intervention	47 OW postpartum (0M, 47W)	31.8 (4.5)*	31.8 (4.0)	NR	Sweden	Parallel	DA, text messages and phone calls	Nordic Nutrition Recommendations 2004	50-60:<30:10-20	Negative	BW, BMI, WC	12	A
	Control	53 OW postpartum (0M, 53W)	32.6 (4.7)*	31.6 (3.4)	NR			DA only	General healthy eating	NR				
Due et al. 2008	Intervention	48 OW/OB (~21M, 27W)	27.3 (4.9)*	31.6 (2.7)*	2.78 (0.81)	Denmark	Parallel	Suppl, DA	Nordic Nutrition Recommendations 2004	60:25:15	Neutral	Glucose, Insulin, LDL-C, **Non-HDL-C, HDL-C TG, BW, BMI WC, CRP	~24	A, I
	Control	25 OW/OB (~11M, 14W)	27.6 (5.1)*	31.3 (2.5)*	2.71 (0.71)				Average Danish Diet	50:35:15				

"IHD, Ischemic Heart Disease; A, agency; C, carbohydrate; DA, dietary advice; F, fat; HC, hypercholesterolemia; I, industry; M, men; MetS, metabolic syndrome; ND, Nordic diet intervention; NR, not reported; OB, obese; OW, overweight; P, protein; Suppl, supplemental feeding control; W, women; BW, body weight; WC, waist circumference; TG, triglycerides; ApoB, apoprotein B; SBP and DBP, systolic and diastolic blood pressure; CRP, c-reactive protein.
a Supplemental feeding control (Supp) is the provision of some meals and foods consumed during the study. Dietary advice (DA) is the provision of counseling on the appropriate intervention and control diets.
b Planned macronutrient composition of intervention and control diets.
c Negative energy balance refers to a deficit in normal energy intake and/or intake below energy requirements. Neutral energy balance refers to the maintenance of usual energy intake and/or meeting energy requirements.
d For ROB, an assessment was performed using the Cochrane Risk of Bias tool, including the evaluation of individual domains of risk of bias (sequence generation, allocation concealment, blinding of participants/ personnel and outcome assessors, incomplete outcome data, selective outcome reporting). Each of the 5 domains was evaluated as either low, high or unclear ROB and the overall ROB category was determined based on the most selected category.
e Agency funding is that from government, university, or not-for-profit sources. Industry funding is that from trade organizations that obtain revenue from the sale of products.
* Calculated before dropout
** Non-HDL-C calculated
†Finland, Sweden, Denmark, Iceland

ESM Table 11. Selected sensitivity analyses in which the systematic removal of an individual trial altered the significance of the effect estimate or the evidence for heterogeneity.

Outcome	Removal of	MD [95% CI], P_{MD}<br>I^2, P_Q
**BLOOD LIPIDS**		
**LDL-C, (mmol/l)**	Adamsson et al. 2010[b,c]	-0.10 [-0.19, -0.02], P_{MD}=0.02<br>88%, P_Q=0.64
	Due et al. 2008[c]	-0.29 [-0.61, 0.02], P_{MD}=0.06<br>92%, P_Q<0.001
	Poulsen et al. 2014[c]	-0.61 [-1.66, -0.45], P_{MD}=0.26<br>57%, P_Q=0.1
	Uusitupa et al. 2013[c]	-0.30 [-0.65, 0.05], P_{MD}=0.10<br>92%, P_Q<0.001
**Non-HDL-C, (mmol/l)**	Adamsson et al. 2010[b,c]	-0.14 [-0.48, 0.20], P_{MD}=0.42<br>0%, P_Q=1
**HDL-C, (mmol/l)**	Adamsson et al. 2010[b]	-0.00 [-0.04, 0.04], P_{MD}=0.92<br>32%, P_Q=0.22
**Triglycerides, (mmol/l)**	Adamsson et al. 2010[a,b]	-0.09 [-0.16, -0.01], P_{MD}=0.02<br>0%, P_Q=0.4
	Poulsen et al. 2014[c]	-0.01 [-0.09, 0.08], P_{MD}=0.90<br>0%, P_Q=0.52
**Apo-B, (g/l)**	Adamsson et al. 2010[c]	-0.04 [-0.10, 0.02], P_{MD}=0.19<br>n/a
**ADIPOSITY**		
**BMI, (kg/m^2)**	Due et al. 2008[b]	-1.04 [-1.27, -0.82], P_{MD}=0.008<br>0%, P_Q=0.60
**Waist circumference, (cm)**	Poulsen et al. 2014[c]	-0.61 [-1.66, -0.45], P_{MD}=0.26<br>57%, P_Q=0.1
	Gotfredsen et al. 2020[b]	-2.49 [-3.66, -1.33], P_{MD}<0.001<br>0%, P_Q=0.60
**BLOOD PRESSURE**		
**Diastolic blood pressure, (mmHg)**	Gotfredsen et al. 2020[b]	-2.32 [-3.83, -0.82], P_{MD}=0.39<br>0%, P_Q=0.002
	Poulsen et al. 2014[b,c]	-1.02 [-2.29, 0.25], P_{MD}=0.11<br>0%, P_Q=0.37
**INFLAMMATION**		
**CRP, (nmol/l)**	Poulsen et al. 2014[b]	-0.02 [-0.43, 0.39], P_{MD}=0.92<br>0%, P_Q=0.51

CRP, c-reactive protein; MD, mean difference
[a] removal of this study results in significance of the overall effect
[b] removal of this study explains heterogeneity
[c] removal of this study results in a loss of significance of the overall effect

ESM Table 12. GRADE assessment for the association between Nordic dietary patterns and cardiometabolic disease outcomes for prospective cohort studies.

Outcome	Cohort comparisons, *n*	Risk of bias	Inconsistency	Indirectness	Imprecision	Other considerations	RR (95% CI)	Certainty	Interpretation of the magnitude of the association
**CVD incidence**	3	not serious	serious[a]	not serious	serious[b]	dose response gradient[c]	0.93 (0.88, 0.99)[l]<br>0.93 (0.88, 0.99)[m]	⊕⊕◯◯<br>LOW	Small important
**CVD mortality**	8	not serious	not serious	not serious	not serious	dose response gradient[d]	0.81 (0.73, 0.90)[l]<br>0.74 (0.69, 0.80)[m]	⊕⊕⊕◯<br>MODERATE	Moderate
**CHD**	5	not serious	not serious[e]	not serious	serious[f]	dose response gradient[g]	0.88 (0.72, 1.06)[l]<br>0.88 (0.79, 0.98)[m]	⊕⊕◯◯<br>LOW	Small important
**Stroke**	3	not serious	not serious	not serious	serious[h]	dose response gradient[i]	0.88 (0.79, 0.98)[l]<br>0.87 (0.78, 0.97)[m]	⊕⊕◯◯<br>LOW	Small important
**T2D**	6	not serious	not serious	not serious	serious[j]	dose response gradient[k]	0.96 (0.86, 1.06)[l]<br>0.91 (0.84, 0.99)[m]	⊕⊕◯◯<br>LOW	Small important

Cohorts start at low-certainty evidence from which the evidence can be upgraded or downgraded based on prespecified criteria. Criteria to upgrade included a dose-response gradient, large magnitude of the effect (RR ≥2 or RR ≤0.5 and attenuation by plausible confounding. Criteria to downgrade included study limitations (NOS [46]); inconsistency (substantial unexplained inter-study heterogeneity, I^2> 50% and P_Q<0.10); indirectness (presence of factors relating to the population, exposures and outcomes that limit generalizability), imprecision (95% CIs for pooled estimates crossed prespecified MIDs, as shown in the table, and publication bias (significant detection of small-study effects).

a. Downgrade applied due to serious inconsistency (I^2 = 88%, P=0.0002).
b. Downgrade for serious imprecision for CVD incidence, as the 95% CI [0.88, 0.99] overlap with the minimally important difference for clinical benefit (RR=0.95).
c. Upgrade for a dose-response gradient, as the GLST analysis revealed a significant linear inverse relationship between the Nordic diet and incident CVD (P<0.001).
d. Upgrade for a dose-response gradient, as the GLST analysis revealed a significant linear inverse relationship between the Nordic diet and CVD mortality (P<0.001).
e. No downgrade for serious inconsistency for the relation of adherence of the Nordic dietary pattern with CHD incidence, as although there was evidence of substantial heterogeneity (I^2 = 58% (P_Q=0.05).), removal of the Danish Diet, Cancer and Health women cohort explained most of the heterogeneity (I^2=7%, P=0.36) without altering the direction, magnitude or significance of the pooled effect estimate (RR 0.96 [95% CI 0.85, 1.09], P=0.51).
f. Downgrade for serious imprecision for CHD incidence, as the 95% CI [0.72, 1.06] overlap with the minimally important difference for clinical benefit (RR=0.95) and harm (RR=1.05).
g. Upgrade for a dose-response gradient, as the GLST analysis revealed a significant linear inverse relationship between the Nordic diet and incident CHD (P<0.001)
h. Downgrade for serious imprecision for stroke incidence, as the 95% CI [0.79, 0.98] overlap with the minimally important difference for clinical benefit (RR=0.95).
i. Upgrade for a dose-response gradient, as the GLST analysis revealed a significant linear inverse relationship between the Nordic diet and incident stroke (P<0.001).
j. Downgrade for serious imprecision for T2D incidence, as the 95% CI [0.86, 1.06] overlap with the minimally important difference for clinical benefit (RR=0.95) and harm (RR=1.05).
k. Upgrade for a dose-response gradient, as the GLST analysis revealed a significant linear inverse relationship between the Nordic diet and incident stroke (P<0.001).
l. Extreme quantiles.
m. Global dose-response meta-analysis (DRM) estimates.

ESM Table 13. NutriGRADE Assessment for association of Nordic dietary patterns with cardiometabolic outcomes in Cohort studies.

		Quality assessment (points; Max 10)								Effect	Score
**Outcome**	**Cohort comparisons, *n*** ***[Number of events/Participants]***	**Risk of bias, study quality and study limitations** *Assessed using NOS as shown in table 6*	**Precision****	**Heterogeneity**	**Directness**	**Publication Bias**	**Funding Bias**	**Effect Size** *Important benefit was defined as RR of <0.8 and harm RR of > 1.2*	**Dose Response**	**Pooled Effect Estimate RR (95% CI)**	**Meta-evidence (Final point)**
**CVD incidence**	3 10,279/60,436	1	1	0	1	0	1	0	1	0.93 (0.88, 0.99)	5 Low
**Reasons**		Low risk of bias	≥500 participants or ≥500 events were included, and the 95% CI excludes the null value	2-5 studies x multiply by 0		Publication bias not assessed		No point was awarded because effect estimate showed small effect size (RR: 0.80 - 1.20).	1 point was awarded for the dose-response association.		
**CVD mortality**	8 11,146/639,086	1	1	0.5	1	0	1	0	1	0.81 (0.73, 0.90)	5.5 Low
**Reasons**		Low risk of bias	≥500 participants or ≥500 events were included, and the 95% CI excludes the null value	.5 point was awarded for reporting no important heterogeneity (I^2<40%) from 8 cohort comparisons (multiplier: 1).		Publication bias not assessed		No point was awarded because effect estimate showed small effect size (RR: 0.80 - 1.20).	1 point was awarded for the dose-response association.		
**CHD**	5 3960/123,382	1	1	0	1	0	1	0	1	0.88 (0.72, 1.06)	5 Low
**Reasons**		Low risk of bias	≥500 events were included, but 95% CI overlaps the null value and 95% CI excludes important benefit (RR <0.8) or harm (RR >1.2).	2-5 studies x multiply by 0		Publication bias not assessed		No point was awarded because effect estimate showed small effect size (RR: 0.80 - 1.20).	1 point was awarded for the dose-response association.		
**Stroke**	3 3302/122,133	1	1	0.5	1	0	1	0	1	0.88 (0.79, 0.98)	5.5 Low

**Reasons**		Low risk of bias	≥500 participants or ≥500 events were included, and the 95% CI excludes the null value			Publication bias not assessed		No point was awarded because effect estimate showed small effect size (RR: 0.80 - 1.20).	1 point was awarded for the dose-response association.		
**T2DM**	6<br>13,121/112,157	1	1	0.2	1	0	1	0	1	0.96 (0.86, 1.06)	5.2 Low
**Reasons**		Low risk of bias	≥500 events were included, but 95% CI overlaps the null value and 95% CI excludes important benefit (RR <0.8) or harm (RR >1.2).	0.1 score each for reporting I2, random effects and multiplier 1 for 6-9 studies.		Publication bias not assessed		No point was awarded because effect estimate showed small effect size (RR: 0.80 - 1.20).	1 point was awarded for the dose-response association.		

Reference: Schwingshackl L, Knüppel S, Schwedhelm C, Hoffmann G, Missbach B, Stelmach-Mardas M, Dietrich S, Eichelmann F, Kontopantelis E, Iqbal K, Aleksandrova K, Lorkowski S, Leitzmann MF, Kroke A, Boeing H. Perspective: NutriGrade: A Scoring System to Assess and Judge the Meta-Evidence of Randomized Controlled Trials and Cohort Studies in Nutrition Research. Adv Nutr 2016;7:994-

ESM Table 14. GRADE assessment for the effect of Nordic dietary patterns and cardiometabolic risk factors in RCTs.

			Quality assessment							
			Downgrades							
Outcome	Studies	Study design	Risk of bias	Inconsistency	Indirectness	Imprecision	Publication Bias[a]	Effect (MD [95%CI], P_{MD})	Interpretation of magnitude of effect[b]	Quality[c]
**Blood lipids**										
LDL-C, mmol/L	5	RCTs	not serious	serious[1]	not serious	serious[2]	not serious	-0.26 [-0.52, -0.00], p=0.050	Small important effect	LOW
Non-HDL-C, mmol/L	4	RCTs	not serious	serious[3]	not serious	not serious	not serious	-0.69 [-0.90, -0.48], p <0.0001	Large effect	MODERATE
HDL-C, mmol/L	5	RCTs	not serious	not serious[4]	not serious	not serious	not serious	-0.03 [-0.10, 0.03], p=0.35	No effect	HIGH
Triglycerides, mmol/L	5	RCTs	not serious	not serious	not serious	serious[5]	not serious	-0.05 [-0.14, 0.05], p=0.34	No effect	MODERATE
ApoB, g/L	2	RCTs	not serious	serious[6]	not serious[7]	not serious	not serious	-0.15 [-0.19, -0.11], p<0.0001	Moderate effect	MODERATE
**Glycemic control**										
HbA1c, %	1	RCTs	not serious	not serious[8]	serious[9]	not serious	not serious	0.01 [-0.06, 0.08], p=0.79	No effect	MODERATE
Fasting glucose, mmol/L	5	RCTs	not serious	not serious	not serious	not serious	not serious	-0.04 [-0.10, 0.02], p=0.46	No effect	HIGH
Fasting insulin, pmol/L	4	RCTs	not serious	not serious	not serious	serious[10]	not serious	-7.83 [-12.26, -3.39], p=0.0005	Small important effect	MODERATE
**Adiposity**										
Body weight, kg	6	RCTs	not serious	serious[11]	not serious	not serious	not serious	-2.00 [-3.24, -0.75], p=0.002	Moderate effect	MODERATE
BMI, kg/m^2	4	RCTs	not serious	not serious	not serious	not serious	not serious	-0.98 [-1.19, -0.77], p<0.0001	Small important effect	HIGH
Waist circumference, cm	4	RCTs	not serious	not serious[12]	not serious	serious[13]	not serious	-1.32 [-2.20, -0.43], p=0.003	Trivial effect	MODERATE
**Blood pressure**										
Systolic, mmHg	4	RCTs	not serious	not serious	not serious	serious[14]	not serious	-3.35 [-5.12, -1.59], p=0.0002	Small important effect	MODERATE
Diastolic, mmHg	4	RCTs	not serious	not serious	not serious	serious[15]	not serious	-1.50 [-2.62, -0.37], p=0.009	Trivial effect	MODERATE
**Inflammation**										
CRP, nmol/L	5	RCTs	not serious	not serious[16]	not serious	serious[17]	not serious	-1.91 [-6.37, 2.55], p=0.4	No effect	MODERATE

Apo-B, apolipoprotein-B; BMI, body mass index; CI, confidence interval; CRP, c-reactive protein; HbA1c, hemoglobin A1c; LDL-C, low density lipoprotein cholesterol; HDL-C, high density lipoprotein cholesterol; MD, mean difference; N/A, not applicable; Non-HDL-C, non-high density lipoprotein cholesterol; RCTs, randomized controlled trials, Small important: quantitative small but important association; Trivial: quantitative small but biologically/clinical unimportant association

[a] No downgrades were made for publication bias, as publication bias could not be assessed due to lack of power for assessing funnel plot asymmetry and small study effects (<10 trials included in the meta-analysis).

[b] We used prespecified MIDs to interpret the magnitude of the effect of the pooled estimate with effect size language defined by GRADE. MIDs for RCT outcomes were: 0.1 mmol/L for LDL-C, non-HDL-C, HDL-C, and TG [50-52]; 0.04 g/L for ApoB; 0.3% for HbA1c; 0.5 mmol/L for fasting blood glucose[53], 5 pmol/L for fasting insulin; 0.5 kg for body weight[54, 55]; 0.2 kg/m2 for BMI; 2 cm for WC; 2 mmHg for SBP and DBP[56]; and 0.5 mg/L for CRP[57, 58]]), and publication bias (significant detection of small-study effects)

[c] Since all included trials were randomized controlled trials, the certainty of the evidence was graded as high for all outcomes by default and then downgraded or upgraded based on pre-specified criteria. Criteria for downgrades included risk of bias (downgraded if the majority of trials were considered to be at high risk of bias by the Cochrane ROB tool); inconsistency (downgraded if there was substantial unexplained heterogeneity [$I^2 \geq 50\%$, $pQ < 0.10$]; indirectness (downgraded if there were factors absent or present relating to the participants, interventions, or outcomes that limited the generalizability of the results); imprecision (downgraded if the 95% confidence interval crossed the minimally important difference [MID] as in b; and publication bias (downgraded if there is evidence of publication bias based on funnel plot asymmetry and/or significant Egger's or Begg's tests ($p<0.10$) with confirmation by adjustment by Duval and Tweedie trim-and-fill analysis).
[1] Downgrade for serious inconsistency for the effect of Nordic diets on LDL-C, as there was evidence of substantial heterogeneity (I^2 = 89% and P<0.001).
[2] Downgrade for serious imprecision for the effect of Nordic diets on LDL-C, as the 95% CIs (-0.52, -0.00 mmol/L) overlap with the minimally important difference for clinical benefit (0.1 mmol/L).
[3] Downgrade for serious inconsistency for the effect of Nordic diets on Non-HDL-C, as there was evidence of substantial heterogeneity (I^2 = 91% and P<0.001), and although removal of Adamsson et al. explained the heterogeneity (I^2=0%, PQ=1), the magnitude of the pooled effect estimate was decreased and significance was lost (MD=-0.14mmol/L, 95% CI:-0.48, 0.20, P=0.42).
[4] No downgrade for serious inconsistency for the effect of Nordic diets on HDL-C, as although there was evidence of substantial heterogeneity(I^2 = 80% and P=0.0005), removal of Adamsson et al. explained the heterogeneity (I^2=32%, P=0.22), without altering the direction, magnitude, or significance of the pooled effect estimate (MD=-0.00mmol/L, 95% CI: -0.04, 0.04, P=0.92)..
[5] Downgrade for serious imprecision for the effect of Nordic diets on triglycerides, as the 95% CIs (-0.14, 0.05 mmol/L) overlap with the minimally important difference for clinical benefit (0.1 mmol/L).
[6] Downgrade for serious inconsistency for the effect of Nordic diets on ApoB as there was evidence of substantial heterogeneity (I^2 = 96% and P<0.001).
[7] No downgrade for indirectness for the effect of Nordic dietary patterns on apoB, as although there were only 2 small trials which may not have been representative, the direction, magnitude of the effect was similar to that of other related apolipoprotein-containing particles, non-HDL-C and LDL-C, both of which demonstrated significant reductions. in 4 and 5 trials, respectively,.
[8] Inconsistency could not be assessed as only one trial comparison was available.
[9]Downgrade for serious indirectness for the effect of Nordic dietary patterns on HbA1c, as only 1 trial comparison was available so replication of the results across different trial conditions and Nordic dietary patterns cannot be confirmed.
[10] Downgrade for serious imprecision for the effect of Nordic diets on fasting insulin as the 95% CIs (-12.26, -3.39pmol/L) overlap with the minimally important difference for clinical benefit (5 pmol/L).
[11] Downgrade for serious inconsistency for the effect of Nordic diets on body weight as there was evidence of substantial heterogeneity (I^2 = 88% and P<0.001).
[12] No downgrade for serious inconsistency for the effect of Nordic diets on waist circumference, as although there was evidence of substantial heterogeneity (I^2=71% and PQ=0.02), removal of Gotfredsen et al. 2020 explained the heterogeneity (I2=0%, PQ=0.60) without altering the direction, magnitude, or significance of the pooled effect estimate (MD=-2.49 cm, 95% CI: -3.66, -1.33, PMD<0.001).
[13] Downgrade for serious imprecision for the effect of Nordic diets on waist circumference as the 95% CIs (-3.36, -1.09cm) overlap with the minimally important difference for clinical benefit (2cm).
[14] Downgrade for serious imprecision for the effect of Nordic diets on systolic blood pressure as the 95% CIs (-5.12, -1.59 mmHg) overlap with the minimally important difference for clinical benefit (2mmHg).
[15] Downgrade for serious imprecision for the effect of Nordic diets on diastolic blood pressure as the 95% CIs (-2.62, -0.37 mmHg) overlap with the minimally important difference for clinical benefit (2mmHg).
[16] No downgrade for serious inconsistency for the effect of Nordic dietary patterns on CRP, as although there was evidence of substantial heterogeneity (I^2 = 69% and P=0.01), removal of Poulsen et al. explained the heterogeneity (I^2=0%, P=0.51), without altering the direction, magnitude, or significance of the pooled effect estimate (MD=0.02nmol/L, 95% CI:-0.43, 0.39nmol/L, PMD=0.92).
[17] Downgrade for serious imprecision for the effect of Nordic diets on CRP as the 95% CIs (-6.37, 2.55nmol/L) overlap with the minimally important difference for clinical benefit (4.8nmol/L).

ESM Table 15. NutriGRADE assessment for the effect of Nordic dietary patterns and cardiometabolic risk factors in RCTs

			Quality assessment (points; Max 10)							Effect	Score
**Outcome**	**Trial comparisons, *n***	**Trial size**	**Risk of bias, study quality and study limitations<br><br>Based upon ROB from suppl. Figure 6**	**Precision**	**Heterogeneity**	**Directness**	**Publication Bias**	**Funding Bias**	**Study design**	**Pooled Effect Estimate RR (95% CI)**	**Meta-evidence (Final point)**
**LDL-C**	5	606	2	0	0	0	0	0	2	-0.26 [-0.52, -0.00]	4<br>Low
				400-2000 participants but 95% CI includes null value	2-5 studies x multiply by 0	Surrogate markers	Publication bias not assessed	At least one author has conflict interest or industry funding			
**Non-HDL-C**	4	374	2	0	0	0	0	0	2	-0.69 [-0.9-, -0.48]	4<br>Low
				<400 participants	2-5 studies x multiply by 0	Surrogate markers	<5 studies	At least one author has conflict interest or industry funding			
**HDL-C**	5	606	2	0	0	0	0	0	2	-0.03 [-0.10, 0.03]	4<br>Low
				400-2000 participants but 95% CI includes null value	2-5 studies x multiply by 0	Surrogate markers	Publication bias not assessed	At least one author has conflict interest or industry funding			
**Triglycerides**	5	606	2	0	0	0	0	0	2	-0.05 [-0.14, 0.05]	4<br>Low
					2-5 studies x multiply by 0	Surrogate markers	Publication bias not assessed	At least one author has conflict interest or industry funding			
**ApoB**	3	262	2	0	0	0	0	0	2	-0.15 [-0.19, -0.11]	4<br>Low
				<400 participants	2-5 studies x multiply by 0	Surrogate markers	<5 studies	At least one author has conflict interest or industry funding			
**HbA_{1c}**	1	145	2	0	0	0	0	1	2	0.01[-0.06,0.08]	5<br>Low
				<400 participants	Only 1 study, No chi2 performed	Surrogate markers	<5 studies				
**Fasting glucose**	5	706	2	0	0	0	0	0	2	-0.04 [-0.10, 0.02]	4<br>Low
				400-2000 participants but 95% CI includes null value	2-5 studies x multiply by 0	Surrogate markers	Publication bias not assessed	At least one author has conflict interest or industry funding			

**Fasting insulin**	4	393	2	0	0	0	0	0	2	-7.83 [-12.26, -3.39]	4<br>Low
				<400 participants	2-5 studies x multiply by 0	Surrogate markers	<5 studies	At least one author has conflict interest or industry funding			
**Body weight**	6	706	2	1	0.2	0	0	0	2	-2.00 [-3.24, -0.75]	5.5<br>Low
					I2>40%	Surrogate markers	Publication bias not assessed	At least one author has conflict interest or industry funding			
**BMI**	4	393	2	0	0	0	0	0	2	-0.98 [-1.19, -0.77]	4<br>Low
				<400 participants	2-5 studies x multiply by 0	Surrogate markers	<5 studies	At least one author has conflict interest or industry funding			
**Waist circumference**	4	454	2	1	0	0	0	0	2	-1.32 [-2.20, -0.43]	5<br>Low
					2-5 studies x multiply by 0	Surrogate markers	<5 studies	At least one author has conflict interest or industry funding			
**Systolic blood pressure**	4	533	2	1	0	0	0	0	2	-3.35 [-5.12, -1.59]	5<br>Low
					2-5 studies x multiply by 0	Surrogate markers	<5 studies	At least one author has conflict interest or industry funding			
**Diastolic blood pressure**	4	533	2	1	0	0	0	0	2	-1.50 [-2.62, -0.37]	5<br>Low
					2-5 studies x multiply by 0	Surrogate markers	<5 studies	At least one author has conflict interest or industry funding			
**CRP**	5	606	2	0	0	0	0	0	2	-1.91 [-6.37, 2.55]	4<br>Low
				400-2000 participants but 95% CI includes null value	2-5 studies x multiply by 0	Surrogate markers	Publication bias not assessed	At least one author has conflict interest or industry funding			

Reference: Schwingshackl L, Knüppel S, Schwedhelm C, Hoffmann G, Missbach B, Stelmach-Mardas M, Dietrich S, Eichelmann F, Kontopantelis E, Iqbal K, Aleksandrova K, Lorkowski S, Leitzmann MF, Kroke A, Boeing H. Perspective: NutriGrade: A Scoring System to Assess and Judge the Meta-Evidence of Randomized Controlled Trials and Cohort Studies in Nutrition Research. Adv Nutr 2016;7:994-

SUPPLEMENTARY FIGURES

ESM Fig. 1. Forest plot of the association between the Nordic dietary patterns and CVD incidence.
RR, risk ratio. Pooled risk estimate is represented by the diamond. Values of $I^2 \geq 50\%$ indicate substantial heterogeneity. Values greater than 1.0 indicate a harmful association.

Cohort	Participants	Cases	Weight	RR, Random, 95% CIs	RR, Random, 95% CIs on CVD incidence
Malmö Diet and Cancer cohort - Males [Hlebowicz et al.,2013]	6,940	1,093	8.20%	0.68 [0.56, 0.83]	
Malmö Diet and Cancer cohort - Females [Hlebowicz et al.,2013]	10,186	703	6.80%	0.73 [0.59, 0.91]	
Swedish Women's Lifestyle and Health Cohort [Roswall et al., 2015(93)]	43,310	8,383	85.00%	0.98 [0.92, 1.04]	
**Total (95% CI)**	**60,436**	**10,179**	**100.00%**	**0.93 [0.88, 0.99]**	

Heterogeneity: $Tau^2 = 0.05$; $Chi^2 = 17.32$, df = 2 (P = 0.0002); $I^2 = 88\%$

Test for overall effect: Z = 2.44 (P = 0.01)

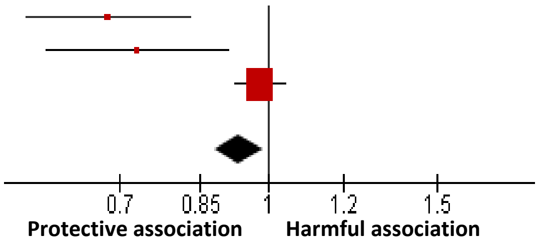

ESM Fig. 2. Forest plot of the association between the Nordic dietary patterns and CVD mortality.

RR, risk ratio. Pooled risk estimate is represented by the diamond. Values of $I^2 \geq 50\%$ indicate substantial heterogeneity. Values greater than 1.0 indicate a harmful association.

Cohort	Participants	Cases	Weight	RR, Random, 95% CIs
EPIC [95]	451,256	3,761	31.6%	0.82 [0.74, 0.90]
Kuopio Ischaemic Heart Disease Risk Factor Study [Tertsunen et al, 2020]	1,547	250	7.5%	0.71 [0.5.1.01]
Malmo Diet and Cancer cohort - Men [Drake et al.,2013]	6,940	444	9.4%	0.59 [0.43, 0.80]
Malmo Diet and Cancer cohort - Women [Drake et al.,2013]	10,186	265	7.3%	1.07 [0.75, 1.53]
Swedish Mammography Cohort [Lemming et al.,2018]	33,341	3,003	24.3%	0.91 [0.79, 1.05]
Swedish Women's Lifestyle and Health Cohort [Roswall et al.,2015(92)]	44,961	270	7.4%	0.88 [0.62, 1.25]
The Copenhagen General Population Study [Ewers et al., 2016]	88,818	2,982	7.5%	0.71 [0.5,1.01]
Western Norway B-vitamin Intervention Trial (WENBIT) [Pauschitz et al. , 2019]	2,019	171	4.9%	0.71 [0.45, 1.12]
**Total (95% CI)**	**639,068**	**11,146**	**100.00%**	**0.81 [0.73, 0.90]**

Heterogeneity: Tau² = 0.01; Chi² = 10.48, df = 7 (P = 0.16); I² = 33%

Test for overall effect: Z = 3.85 (P = 0.0001)

RR, Random, 95% CIs on CVD mortality

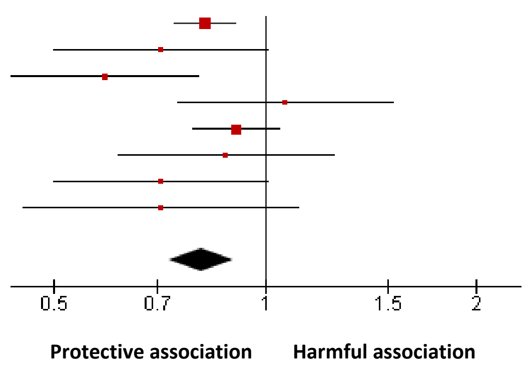

ESM Fig. 3. Forest plot of the association between the Nordic dietary patterns and CHD incidence.

RR, risk ratio. Pooled risk estimate is represented by the diamond. Values of $I^2 \geq 50\%$ indicate substantial heterogeneity. Values greater than 1.0 indicate a harmful association.

Cohort	Participants	Cases	Weight	RR, Random, 95% CIs
Danish Diet, Cancer and Health cohort - Male [Gunge et al.,2017]	25,759	1,669	23.9%	0.86 [0.69, 1.08]
Danish Diet, Cancer and Health cohort - Females [Gunge et al.,2017]	28,809	653	13.7%	0.56 [0.37, 0.84]
EPIC-Potsdam [Galbete et al, 2018]	23,485	312	18.1%	0.88 [0.64, 1.20]
Swedish Women's Lifestyle and Health Cohort [Roswall et al, 2015]	43,310	1,019	27.0%	1.09 [0.91, 1.31]
Western Norway B-vitamin Intervention Trial (WENBIT) [Pauschitz et al. , 2019]	2,019	307	17.4%	0.90 [0.65, 1.25]
**Total (95% CI)**	**123,382**	**3,960**	**100.00%**	**0.88 [0.72, 1.06]**

Heterogeneity: Tau² = 0.03; Chi² = 9.57, df = 4 (P = 0.05); I² = 58%

Test for overall effect: Z = 1.38 (P = 0.17)

RR, Random, 95% CIs on CHD incidence

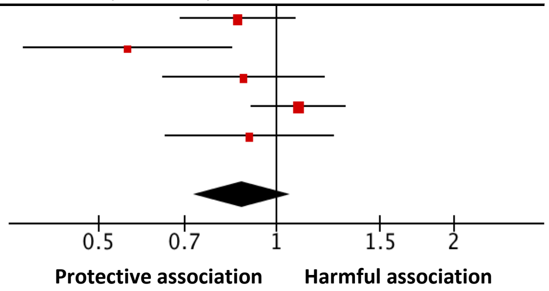

ESM Fig. 4. Forest plot of the association between the Nordic dietary patterns and stroke incidence.
RR, risk ratio. Pooled risk estimate is represented by the diamond. Values of $I^2 \geq 50\%$ indicate substantial heterogeneity. Values greater than 1.0 indicate a harmful association.

Cohort	Participants	Cases	Weight	RR, Fixed, 95% CIs
Danish Diet, Cancer and Health cohort [Hansen et al.,2017]	55,338	2,283	65.1%	0.83 [0.73, 0.95]
EPIC-Potsdam [Galbete et al, 2018]	23,485	321	12.3%	0.97 [0.72, 1.31]
Swedish Women's Lifestyle and Health Cohort [Roswall et al, 2015]	43,310	698	22.6%	0.98 [0.78, 1.23]
**Total (95% CI)**	**122,133**	**3,302**	**100.0%**	**0.88 [0.79, 0.98]**
**Heterogeneity: Tau² = 0.00; Chi² = 2.04, df = 2 (P = 0.36); I² = 2%**				
**Test for overall effect: Z = 2.32 (P = 0.02)**				

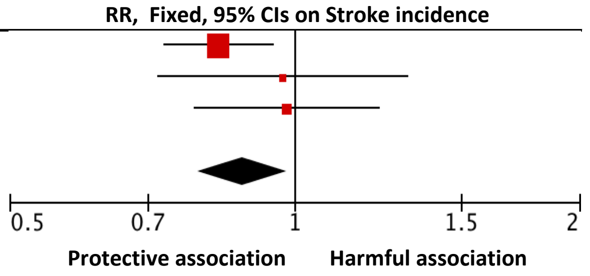

ESM Fig. 5. Forest plot of the association between the Nordic dietary patterns and type 2 diabetes mellitus incidence.
LogRR, logarithmic risk ratio. Pooled risk estimate is represented by the diamond. Values of $I^2 \geq 50\%$ indicate substantial heterogeneity. Values greater than 1.0 indicate a harmful association.

Cohort	Participants	Cases	Weight	RR, Random, 95% CIs
Danish Diet, Cancer and Health cohort - Men [Lacoppidan et al.,2015]	26,107	4,097	19.9%	0.80 [0.69, 0.93]
Danish Diet, Cancer and Health cohort - Women [Lacoppidan et al.,2015]	28,953	3,269	14.2%	0.89 [0.72,1.10]
EPIC-Postdam [Galbete et al, 2018]	23,485	1,376	20.1%	1.01 [0.87, 1.18]
Helsinki Birth Cohort Study, Health 2000 Survey [Karneva et al.,2014]	6,744	541	10.9%	0.93 [0.72, 1.21]
Malmo Diet Study - Men [Mandalazi et al.,2016]	10,413	1,859	16.1%	1.02 [0.84, 1.23]
Malmo Diet Study - Women [Mandalazi et al.,2016]	16,455	1,979	18.8%	1.10 [0.93,1.30]
**Total (95% CI)**	**112,157**	**13,121**	**100.00%**	**0.96 [0.86, 1.06]**
**Heterogeneity: Tau² = 0.01; Chi² = 9.35, df = 6 (P = 0.1); I² = 47%**				
**Test for overall effect: Z = 0.88 (P = 0.38)**				

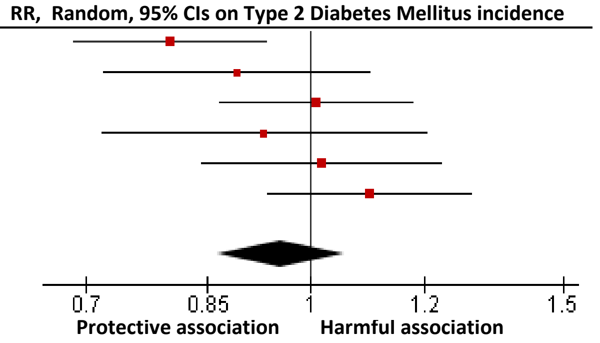

ESM Fig. 6. Risk of bias of included RCTs.
Colored bars represent the proportion of studies assessed and circles represent the individual RCT. The colors represent low (green), unclear (yellow) or high (red) risk of bias for the 5 domains of bias above according to criteria set by the Cochrane Risk of Bias tool.

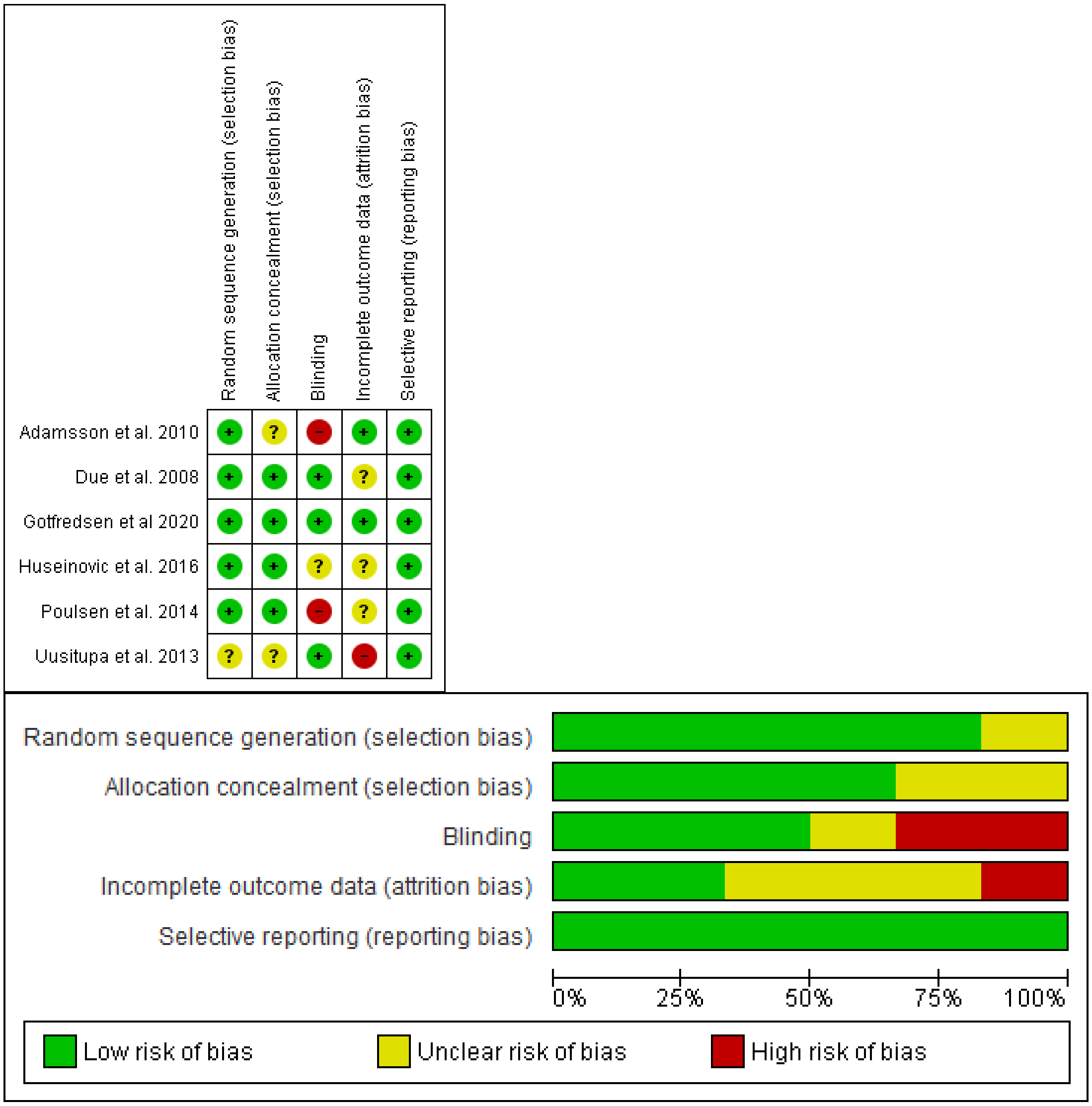

ESM Fig. 7. Forest plot of randomized controlled trials assessing the effect of Nordic diets on LDL-C.

Risk of bias: A=random sequence generation; B=allocation concealment; C=blinding of participants and personnel; D=incomplete outcome data; E=selective reporting. Pooled effect estimates for each subgroup and overall effect are represented by the diamonds. Data are expressed as weighted mean differences with 95% CIs using the generic inverse-variance method modelled by random effects. Paired analyses were applied to all crossover trials. Inter-study heterogeneity was assessed using the Cochran Q statistic and quantified using the I^2 statistic, with significance set at $P<0.10$ and $I^2>50\%$ considered to be evidence of substantial heterogeneity.

Study or Subgroup	Participants		Weight	Mean Difference
	Nordic Diet	Control		IV, Random, 95% CIs
Adamsson et. al. 2010	44	42	18.7%	-0.93 [-1.18, -0.68]
Due et al. 2008	48	25	18.1%	-0.13 [-0.41, 0.15]
Gotfredsen et al. 2020	72	73	21.1%	-0.02 [-0.08, 0.06]
Poulsen et al 2014	91	56	21.0%	-0.14 [-0.30, 0.02]
Uusitupa et al 2013	96	70	21.0%	-0.15 [-0.31, 0.01]
**Total (95% CI)**			**100.0%**	**-0.26 [-0.52,- 0.00]**

Heterogeneity: Tau²=0.08, Chi² = 37.84, df = 4 (P< 0.00001); I² = 89%
Test for overall effect: Z = 1.97 (P = 0.05)

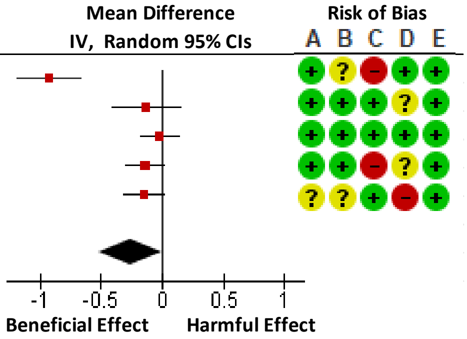

ESM Fig. 8. Forest plot of randomized controlled trials assessing the effect of Nordic dietary patterns on BMI.

Risk of bias: A=random sequence generation; B=allocation concealment; C=blinding of participants and personnel; D=incomplete outcome data; E=selective reporting. Pooled effect estimates for each subgroup and overall effect are represented by the diamonds. Data are expressed as weighted mean differences with 95% CIs using the generic inverse-variance method modelled by random effects. Paired analyses were applied to all crossover trials. Interstudy heterogeneity was assessed using the Cochran Q statistic and quantified using the I^2 statistic, with significance set at $P<0.10$ and $I^2>50\%$ considered to be evidence of substantial heterogeneity.

Study or Subgroup	Participants		Weight	Mean Difference
	Nordic Diet	Control		IV, Fixed, 95% CIs
Adamsson et. al. 2010	44	42	82.4%	-1.03 [-1.26, -0.80]
Due et al. 2008	48	52	12.1%	-0.50 [-1.11, 0.11]
Gotfredsen et al. 2020	72	73	0.1%	2.46 [-5.66,10.58]
Huseinovic et al. 2016	47	53	16.3%	-1.30 [-2.21, -0.39]
**Total (95% CI)**			**100.0%**	**-0.98 [-1.19, -0.77]**

Heterogeneity:Chi² =3.69, df =3 (P=0.3); I² = 19%
Test for overall effect: Z = 8.99 (P < 0.00001)

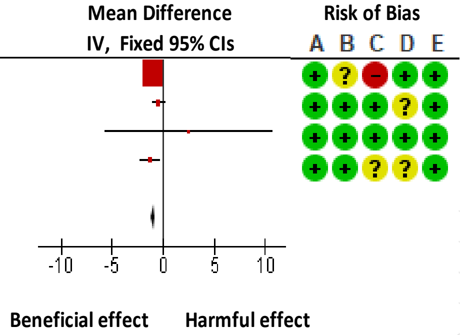

ESM Fig. 9. Forest plot of randomized controlled trials assessing the effect of Nordic dietary patterns on body weight.

Risk of bias: A=random sequence generation; B=allocation concealment; C=blinding of participants and personnel; D=incomplete outcome data; E=selective reporting. Pooled effect estimates for each subgroup and overall effect are represented by the diamonds. Data are expressed as weighted mean differences with 95% CIs using the generic inverse-variance method modelled by random effects. Paired analyses were applied to all crossover trials. Inter-study heterogeneity was assessed using the Cochran Q statistic and quantified using the I2 statistic, with significance set at P<0.10 and I2>50% considered to be evidence of substantial heterogeneity.

Study or Subgroup	Participants Nordic Diet	Control	Weight	Mean Difference IV, Random, 95% CIs	Risk of Bias A	B	C	D	E
Adamsson et. al. 2010	44	42	19.7%	-3.03 [-3.74, -2.32]	+	?	-	+	+
Due et al. 2008	48	52	13.8%	-1.60 [-3.58, 0.38]	+	+	+	?	+
Gotfredsen et al. 2020	72	73	18.6%	-0.71 [-1.69,0.29]	+	+	+	+	+
Huseinovic et al. 2016	47	53	11.1%	-3.70 [-6.26, -1.14]	+	+	?	?	+
Poulsen et al 2014	91	56	16.6%	-3.22 [-4.62, -1.82]	+	+	-	?	+
Uusitupa et al 2013	96	70	20.2%	-0.50 [-1.05, 0.05]	?	?	+	-	+
**Total (95% CI)**			**100.0%**	**-2.00 [-3.24, -0.75]**					

Heterogeneity: Tau²=1.92, Chi² = 41.79, df = 5 (P < 0.0001); I² = 88%
Test for overall effect: Z = 3.14(P = 0.002)

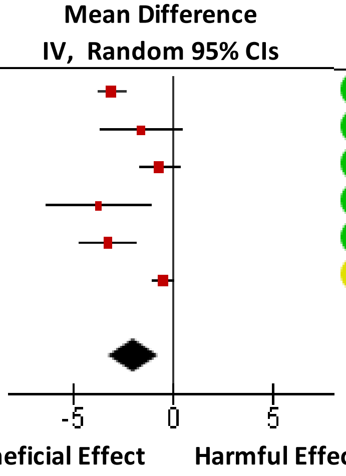

ESM Fig. 10. Forest plot of randomized controlled trials assessing the effect of Nordic dietary patterns on waist circumference.

Risk of bias: A=random sequence generation; B=allocation concealment; C=blinding of participants and personnel; D=incomplete outcome data; E=selective reporting. Pooled effect estimates for each subgroup and overall effect are represented by the diamonds. Data are expressed as weighted mean differences with 95% CIs using the generic inverse-variance method modelled by random effects. Paired analyses were applied to all crossover trials. Inter-study heterogeneity was assessed using the Cochran Q statistic and quantified using the I^2 statistic, with significance set at P<0.10 and I^2>50% considered to be evidence of substantial heterogeneity.

Study or Subgroup	Participants Nordic Diet	Control	Weight	Mean Difference IV, Fixed, 95% CIs	Risk of Bias A	B	C	D	E
Due et al. 2008	48	52	19.0%	-1.40 [-3.89, 1.09]	+	+	+	?	+
Gotfredsen et al. 2020	72	73	27.4%	-0.28 [-1.07,1.63]	+	+	+	+	+
Huseinovic et al. 2016	47	53	20.3%	-2.50 [-4.81, -0.19]	+	+	?	?	+
Poulsen et al 2014	91	56	25.9%	-2.94 [-4.54, -1.34]	+	+	-	?	+
**Total (95% CI)**			**100.0%**	**-1.32 [-2.20, -0.43]**					

Heterogeneity: Chi² = 10.30, df = 3 (P = 0.02); I² = 71%
Test for overall effect: Z = 2.92 (P = 0.003)

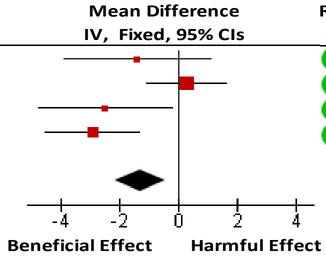

ESM Fig. 11. Forest plot of randomized controlled trials assessing the effect of Nordic dietary patterns on HDL-C.

Risk of bias: A=random sequence generation; B=allocation concealment; C=blinding of participants and personnel; D=incomplete outcome data; E=selective reporting. Pooled effect estimates for each subgroup and overall effect are represented by the diamonds. Data are expressed as weighted mean differences with 95% CIs using the generic inverse-variance method modelled by random effects. Paired analyses were applied to all crossover trials. Inter-study heterogeneity was assessed using the Cochran Q statistic and quantified using the I^2 statistic, with significance set at $P<0.10$ and $I^2>50\%$ considered to be evidence of substantial heterogeneity.

Study or Subgroup	Participants Nordic Diet	Participants Control	Weight	Mean Difference IV, Random, 95% CIs	Risk of Bias A	B	C	D	E
Adamsson et. al. 2010	44	42	18.7%	-0.19 [-0.28, -0.10]	+	?	−	+	+
Due et al. 2008	48	52	11.9%	-0.04 [-0.19, 0.11]	+	+	+	?	+
Gotfredsen et al. 2020	72	73	23.1%	-0.01 [-0.08, 0.06]	+	+	+	+	+
Poulsen et al 2014	91	56	23.7%	-0.03 [-0.08, 0.02]	+	+	−	?	+
Uusitupa et al 2013	96	70	22.5%	-0.05 [-0.01, 0.11]	?	?	+	−	+
**Total (95% CI)**			**100.0%**	**-0.03 [-0.10, 0.03]**					

Heterogeneity: Tau²=0.00, Chi² = 20.02, df = 5 (P = 0.001); I² = 75%
Test for overall effect: Z = 0.93 (P = 0.35)

Mean Difference IV, Random 95% CIs

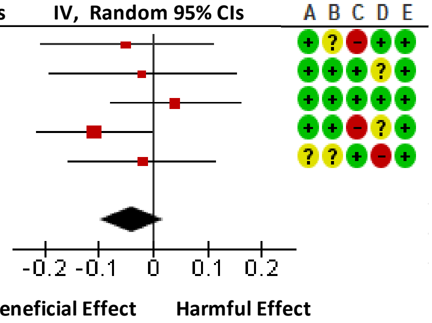

ESM Fig. 12. Forest plot of randomized controlled trials assessing the effect of Nordic dietary patterns on Non-HDL-C.

Risk of bias: A=random sequence generation; B=allocation concealment; C=blinding of participants and personnel; D=incomplete outcome data; E=selective reporting. Pooled effect estimates for each subgroup and overall effect are represented by the diamonds. Data are expressed as weighted mean differences with 95% CIs using the generic inverse-variance method modelled by random effects. Paired analyses were applied to all crossover trials. Inter-study heterogeneity was assessed using the Cochran Q statistic and quantified using the I^2 statistic, with significance set at $P<0.10$ and $I^2>50\%$ considered to be evidence of substantial heterogeneity.

Study or Subgroup	Participants Nordic Diet	Participants Control	Weight	Mean Difference IV, Fixed, 95% CIs	Risk of Bias A	B	C	D	E
Adamsson et. al. 2010	44	42	62.5%	-1.02 [-1.28, -0.76]	+	?	−	+	+
Due et al. 2008	48	52	32.4%	-0.14 [-0.50, 0.22]	+	+	+	?	+
Gotfredsen et al. 2020	72	73	4.2%	-0.13 [-1.14, 0.88]	+	+	+	+	+
Uusitupa et al 2013	96	70	0.8%	-0.18 [-2.52, 2.16]	?	?	+	−	+
**Total (95% CI)**			**100.0%**	**-0.69 [-0.90, -0.48]**					

Heterogeneity: Chi² = 16.18, df = 3 (P = 0.001); I² = 81%
Test for overall effect: Z = 6.51 (P < 0.001)

Mean Difference IV, Fixed 95% CIs

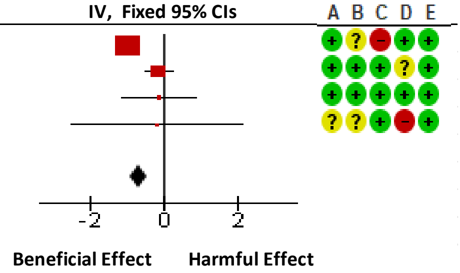

ESM Fig. 13. Forest plot of randomized controlled trials assessing the effect of Nordic dietary patterns on triglycerides.

Risk of bias: A=random sequence generation; B=allocation concealment; C=blinding of participants and personnel; D=incomplete outcome data; E=selective reporting. Pooled effect estimates for each subgroup and overall effect are represented by the diamonds. Data are expressed as weighted mean differences with 95% CIs using the generic inverse-variance method modelled by random effects. Paired analyses were applied to all crossover trials. Inter-study heterogeneity was assessed using the Cochran Q statistic and quantified using the I2 statistic, with significance set at P<0.10 and I2>50% considered to be evidence of substantial heterogeneity.

Study or Subgroup	Participants Nordic Diet	Control	Weight	Mean Difference IV, Random, 95% CIs
Adamsson et. al. 2010	44	42	14.5%	0.14 [-0.07, 0.35]
Due et al. 2008	48	52	18.4%	-0.04 [-0.21, 0.13]
Gotfredsen et al. 2020	72	73	17.1%	-0.04 [-0.22, 0.14]
Poulsen et al 2014	91	56	27.1%	-0.17 [-0.29, -0.05]
Uusitupa et al 2013	96	70	22.7%	-0.03 [-0.17, 0.11]
**Total (95% CI)**			**100.0%**	**-0.05 [-0.14, 0.05]**

Heterogeneity: Tau²=0.01, Chi² = 6.98, df = 4 (P = 0.14); I² = 43%
Test for overall effect: Z = 0.96 (P = 0.34)

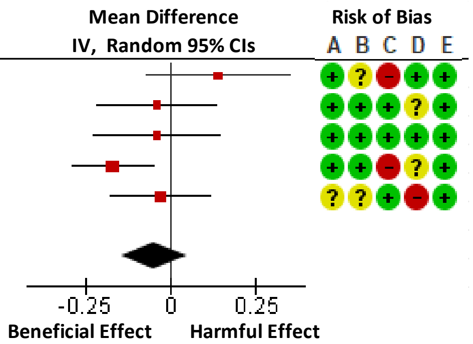

ESM Fig. 14. Forest plot of randomized controlled trials assessing the effect of Nordic dietary patterns on ApoB.

Risk of bias: A=random sequence generation; B=allocation concealment; C=blinding of participants and personnel; D=incomplete outcome data; E=selective reporting. Pooled effect estimates for each subgroup and overall effect are represented by the diamonds. Data are expressed as weighted mean differences with 95% CIs using the generic inverse-variance method modelled by random effects. Paired analyses were applied to all crossover trials. Inter-study heterogeneity was assessed using the Cochran Q statistic and quantified using the I^2 statistic, with significance set at P<0.10 and I^2>50% considered to be evidence of substantial heterogeneity.

Study or Subgroup	Participants Nordic Diet	Control	Weight	Mean Difference IV, Fixed, 95% CIs
Adamsson et. al. 2010	44	42	52.3%	-0.25 [-0.31, -0.19]
Uusitupa et al 2013	96	70	47.7%	-0.04 [-0.10, 0.02]
**Total (95% CI)**			**100.0%**	**-0.15 [-0.19, -0.11]**

Heterogeneity: Chi² = 24.62, df = 1 (P < 0.0001); I² = 96%
Test for overall effect: Z = 7.09 (P < 0.0001)

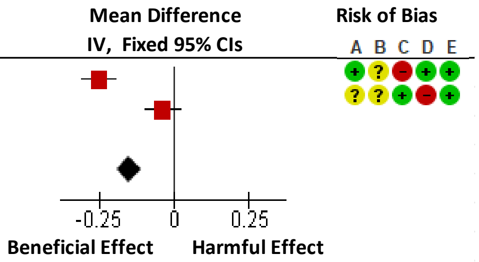

ESM Fig. 15. Forest plot of randomized controlled trials assessing the effect of Nordic dietary patterns on systolic blood pressure.

Risk of bias: A=random sequence generation; B=allocation concealment; C=blinding of participants and personnel; D=incomplete outcome data; E=selective reporting. Pooled effect estimates for each subgroup and overall effect are represented by the diamonds. Data are expressed as weighted mean differences with 95% CIs using the generic inverse-variance method modelled by random effects. Paired analyses were applied to all crossover trials. Inter-study heterogeneity was assessed using the Cochran Q statistic and quantified using the I^2 statistic, with significance set at $P<0.10$ and $I^2>50\%$ considered to be evidence of substantial heterogeneity.

Study or Subgroup	Participants		Weight	Mean Difference	Risk of Bias				
	Nordic Diet	Control		IV, Fixed, 95% CIs	A	B	C	D	E
Adamsson et al. 2010	44	42	11.7%	5.95 [0.78, 11.12]	+	?	−	+	+
Gotfredsen et al. 2020	72	73	31.3%	-1.0 [-4.16,2.16]	+	+	+	+	+
Poulsen et al 2014	91	56	34.0%	-5.13 [-8.16, -2.10]	+	+	−	?	+
Uusitupa et al. 2013	96	70	23.1%	-2.00 [-5.68, 1.68]	?	?	+	−	+
**Total (95% CI)**			**100.0%**	**-3.35 [-5.12, -1.59]**					

Heterogneity: Chi² = 6.04, df = 3 (P = 0.11); I² = 50%
Test for overall effect: Z = 3.72 (P = 0.0002)

Mean Difference IV, Fixed, 95% CIs

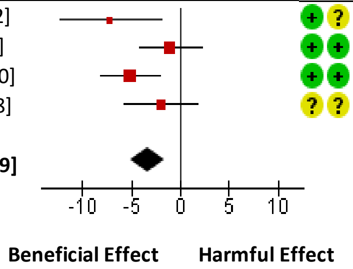

ESM Fig. 16. Forest plot of randomized controlled trials assessing the effect of Nordic dietary patterns on diastolic blood pressure.

Risk of bias: A=random sequence generation; B=allocation concealment; C=blinding of participants and personnel; D=incomplete outcome data; E=selective reporting. Pooled effect estimates for each subgroup and overall effect are represented by the diamonds. Data are expressed as weighted mean differences with 95% CIs using the generic inverse-variance method modelled by random effects. Paired analyses were applied to all crossover trials. Inter-study heterogeneity was assessed using the Cochran Q statistic and quantified using the I^2 statistic, with significance set at $P<0.10$ and $I^2>50\%$ considered to be evidence of substantial heterogeneity.

Study or Subgroup	Participants		Weight	Mean Difference	Risk of Bias				
	Nordic Diet	Control		IV, Fixed, 95% CIs	A	B	C	D	E
Adamsson et al. 2010	44	42	8.3%	-3.47 [-7.36, 0.42]	+	?	−	+	+
Gotfredsen et al.	72	73	44.1%	-0.45 [-2.14, 1.24]	+	+	+	+	+
Poulsen et al 2014	91	56	21.5%	-3.24 [-5.66, -0.82]	+	+	−	?	+
Uusitupa et al. 2013	96	70	26.0%	-1.20 [-3.40, -1.00]	?	?	+	−	+
**Total (95% CI)**			**100.0%**	**-1.50 [-2.62, -0.37]**					

Heterogeneity: Chi² = 4.53, df = 3 (P = 0.21); I² = 34%
Test for overall effect: Z = 2.61(P = 0.009)

Mean Difference IV, Fixed 95% CIs

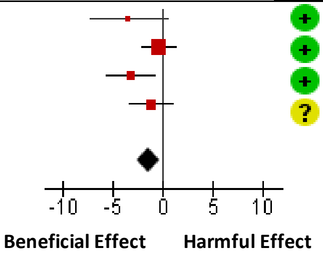

ESM Fig. 17. Forest plot of randomized controlled trials assessing the effect of Nordic dietary patterns on fasting blood glucose.

Risk of bias: A=random sequence generation; B=allocation concealment; C=blinding of participants and personnel; D=incomplete outcome data; E=selective reporting. Pooled effect estimate is represented by the diamond. Data are expressed as weighted mean differences with 95% CIs using the generic inverse-variance method modelled by fixed effects. Paired analyses were applied to all crossover trials. Inter-study heterogeneity was assessed using the Cochran Q statistic and quantified using the I^2 statistic, with significance set at $P<0.10$ and $I^2>50\%$ considered to be evidence of substantial heterogeneity.

Study or Subgroup	Participants Nordic Diet	Participants Control	Weight	Mean Difference IV, Random, 95% CIs	Risk of Bias A	B	C	D	E
Adamsson et. al. 2010	44	42	13.7%	-0.05 [-0.21, -0.11]	+	?	-	+	+
Due et al. 2008	48	52	11.6%	-0.02 [-0.19, 0.15]	+	+	+	?	+
Gotfredsen et al. 2020	72	73	24.0%	0.04 [-0.08 0.16]	+	+	+	+	+
Poulsen et al 2014	91	56	31.8%	-0.11 [-0.21, -0.01]	+	+	-	?	+
Uusitupa et al 2013	96	70	18.9%	-0.02 [-0.15, 0.11]	?	?	+	-	+
**Total (95% CI)**			**100.0%**	**-0.04 [-0.10, 0.02]**					

Heterogeneity: Tau²=0.00,Chi² = 3.59, df = 4 (P 0.46); I² = 0%
Test for overall effect: Z = 1.28 (P = 0.20)

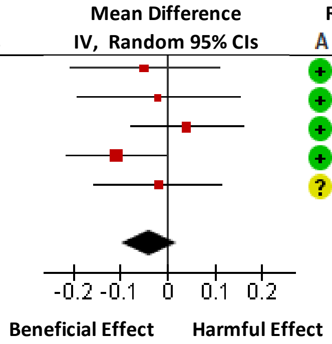

ESM Fig. 18. Forest plot of randomized controlled trials assessing the effect of Nordic dietary patterns on fasting blood insulin.

Risk of bias: A=random sequence generation; B=allocation concealment; C=blinding of participants and personnel; D=incomplete outcome data; E=selective reporting. Pooled effect estimate is represented by the diamond. Data are expressed as weighted mean differences with 95% CIs using the generic inverse-variance method modelled by fixed effects. Paired analyses were applied to all crossover trials. Inter-study heterogeneity was assessed using the Cochran Q statistic and quantified using the I^2 statistic, with significance set at $P<0.10$ and $I^2>50\%$ considered to be evidence of substantial heterogeneity.

Study or Subgroup	Participants Nordic Diet	Participants Control	Weight	Mean Difference IV, Fixed, 95% CIs	Risk of Bias A	B	C	D	E
Adamsson et. al. 2010	44	42	34.0%	-9.79 [-17.40, -2.18]	+	?	-	+	+
Due et al. 2008	48	52	18.4%	-9.70 [-20.03, 0.63]	+	+	+	?	+
Gotfredsen et al. 2020	72	73	21.0%	-1.67 [-11.34, 8.00]	+	+	+	+	+
Poulsen et al 2014	91	56	26.5%	-8.89 [-17.50, -0.28]	+	+	-	?	+
**Total (95% CI)**			**100.0%**	**-7.83 [-12.26, -3.39]**					

Heterogeneity:Chi² = 2.00, df = 3 (P 0.57); I² = 0%
Test for overall effect: Z = 3.46 (P = 0.0005)

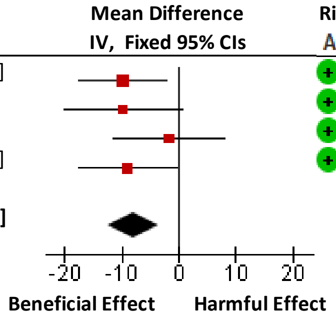

ESM Fig. 19. Forest plot of randomized controlled trials assessing the effect of Nordic dietary patterns on HbA1c.

Risk of bias: A=random sequence generation; B=allocation concealment; C=blinding of participants and personnel; D=incomplete outcome data; E=selective reporting. Pooled effect estimate is represented by the diamond. Data are expressed as weighted mean differences with 95% CIs using the generic inverse-variance method modelled by fixed effects. Paired analyses were applied to all crossover trials. Inter-study heterogeneity was assessed using the Cochran Q statistic and quantified using the I^2 statistic, with significance set at $P<0.10$ and $I^2>50\%$ considered to be evidence of substantial heterogeneity. The overall mean difference (MD) for HbA1c is 0.062 mmol/mol [-0.37, 0.50 mmol/mol] (MD 0.01 %[-0.06, 0.08]).

Study or Subgroup	Participants Nordic Diet	Participants Control	Weight	Mean Difference IV, FIxed, 95% CIs
Gotfredsen et al. 2020	72	73	100.0%	0.01 [-0.06, 0.08]
**Total (95% CI)**			**100.0%**	**0.01 [-0.06, 0.08]**

Heterogeneity: Not applicable
Test for overall effect: Z = 0.26 (P = 0.79)

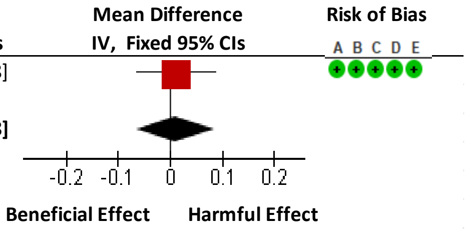

ESM Fig. 20. Forest plot of randomized controlled trials assessing the effect of Nordic dietary patterns on inflammation.

Study or Subgroup	Participants Nordic Diet	Participants Control	Weight	Mean Difference IV, Random, 95% CIs
Adamsson et. al. 2010	44	42	18.0%	-2.19 [-9.80, 5.42]
Due et al. 2008	48	52	9.7%	-4.76 [-17.09, 7.57]
Gotfredsen et al. 2020	72	73	37.5%	-0.03 [-0.44, 0.38]
Poulsen et al 2014	91	56	8.5%	-21.14 [-34.62, -7.67]
Uusitupa et al 2013	96	70	26.3%	2.86 [-1.90, 7.62]
**Total (95% CI)**			**100.0%**	**-1.91 [-6.37, 2.55]**

Heterogeneity: Tau²=13.80, Chi² = 11.72, df = 5 (P = 0.02); I² = 66%
Test for overall effect: Z = 0.84 (P = 0.4)

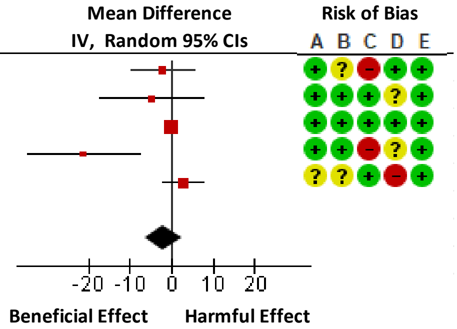

ESM Fig. 21. Influence analysis plots of ad libitum randomized controlled trials assessing the effect of Nordic dietary patterns on adiposity markers.

Influence Analysis
Body Weight

Study Removed	Mean Difference with 95% CI	P_{Effect}	I^2 (%)	$P_{Heterogeneity}$
**Overall**	-2.16 [-3.51 to -0.82]	0.002	82	0.001
Adamsson et al. 2010	-1.80 [-3.46 to -0.14]	0.033	76	0.016
Due et al. 2008	-2.30 [-3.92 to -0.68]	0.005	87	< 0.001
Gotfredsen et al. 2020	-2.93 [-3.53 to -2.33]	< 0.001	0	0.371
Poulsen et al. 2014	-1.82 [-3.54 to -0.11]	0.037	86	< 0.001

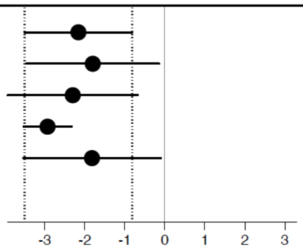

Influence analysis: Removal of each study, one at a time and recalculation of the overall effect and heterogeneity

Influence Analysis
Body Mass Index

Study Removed	Mean Difference with 95% CI	P_{Effect}	I^2 (%)	$P_{Heterogeneity}$
**Overall**	-0.85 [-1.31 to -0.40]	< 0.001	37	0.204
Adamsson et al. 2010	-0.48 [-1.09 to 0.13]	0.122	0	0.476
Due et al. 2008	-1.03 [-1.26 to -0.79]	< 0.001	0	0.400
Gotfredsen et al. 2020	-0.84 [-1.33 to -0.34]	< 0.001	60	0.114

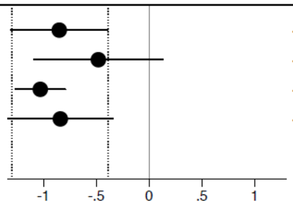

Influence analysis: Removal of each study, one at a time and recalculation of the overall effect and heterogeneity

Influence Analysis
Waist Circumference

Study Removed	Mean Difference with 95% CI	P_{Effect}	I^2 (%)	$P_{Heterogeneity}$
**Overall**	-1.32 [-3.49 to 0.84]	0.233	78	0.010
Due et al. 2008	-1.30 [-4.45 to 1.86]	0.419	89	0.003
Gotfredsen et al. 2020	-2.48 [-3.86 to -1.10]	< 0.001	4	0.308
Poulsen et al. 2014	-0.22 [-1.73 to 1.29]	0.774	26	0.246

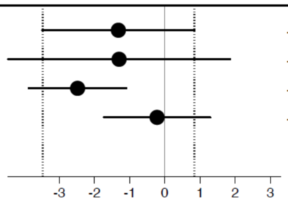

Influence analysis: Removal of each study, one at a time and recalculation of the overall effect and heterogeneity
